# ALS motor neurons exhibit hallmark metabolic defects that are rescued by SIRT3 activation

**DOI:** 10.1038/s41418-020-00664-0

**Published:** 2020-11-12

**Authors:** Jin-Hui Hor, Munirah Mohamad Santosa, Valerie Jing Wen Lim, Beatrice Xuan Ho, Amy Taylor, Zi Jian Khong, John Ravits, Yong Fan, Yih-Cherng Liou, Boon-Seng Soh, Shi-Yan Ng

**Affiliations:** 1 Institute of Molecular and Cell Biology, A*STAR Research Entities, Singapore, 138673 Singapore; 2 Department of Biological Sciences, National University of Singapore, Singapore, 117543 Singapore; 3 Yong Loo Lin School of Medicine (Physiology), National University of Singapore, Singapore, 117456 Singapore; 4 Department of Neurosciences, University of California, San Diego, CA USA; 5 School of Biological Sciences, Nanyang Technological University, Singapore, 637551 Singapore; 6 The Third Affiliated Hospital of Guangzhou Medical University, Guangzhou, 510150 China; 7 National Neuroscience Institute, Singapore, 308433 Singapore

**Keywords:** Neuroscience, Pathogenesis

## Abstract

Motor neurons (MNs) are highly energetic cells and recent studies suggest that altered energy metabolism precede MN loss in amyotrophic lateral sclerosis (ALS), an age-onset neurodegenerative disease. However, clear mechanistic insights linking altered metabolism and MN death are still missing. In this study, induced pluripotent stem cells from healthy controls, familial ALS, and sporadic ALS patients were differentiated toward spinal MNs, cortical neurons, and cardiomyocytes. Metabolic flux analyses reveal an MN-specific deficiency in mitochondrial respiration in ALS. Intriguingly, all forms of familial and sporadic ALS MNs tested in our study exhibited similar defective metabolic profiles, which were attributed to hyper-acetylation of mitochondrial proteins. In the mitochondria, Sirtuin-3 (SIRT3) functions as a mitochondrial deacetylase to maintain mitochondrial function and integrity. We found that activating SIRT3 using nicotinamide or a small molecule activator reversed the defective metabolic profiles in all our ALS MNs, as well as correct a constellation of ALS-associated phenotypes.

## Introduction

Amyotrophic lateral sclerosis (ALS) is an age-onset, progressive neurodegenerative disorder affecting both upper and lower motor neurons (MNs). The loss of MNs leads to denervation of skeletal muscles resulting in muscular atrophy and is eventually fatal. Majority of ALS cases (up to 90%) are sporadic, where the cause of disease is largely unknown. The rest of ALS patients have a familial form of the disease where mutations in genes such as SOD1, C9ORF72, and TDP43 are most common. Despite the genetic differences, the clinical manifestations of sporadic and familial ALS patients are indiscernible, suggesting a possible converging pathogenic mechanism.

In patients, besides MN degeneration, ALS is associated with impairments of energy metabolism, and clinical evidence supports a positive correlation between defective energy metabolism and rate of ALS progression [1]. Animal models of ALS also support metabolic dysregulation as a contributing pathogenic pathway. Previous studies reported that SOD1^G93A^ mice developed progressive central nervous system acidosis as ALS advanced [2], suggesting that neural cell metabolism in ALS is affected. Increasingly, studies have shown that mitochondrial abnormalities are associated with ALS [3–6], with impaired oxidative phosphorylation likely contributing to the ALS pathology [7]. While these collective evidences suggest that mitochondrial dysfunctions are associated with ALS pathology in SOD1 forms of the disease, the mechanisms underlying this observation are not elucidated. In addition, it is not known if metabolic dysfunctions are also a feature of sporadic ALS and non-SOD1 familial ALS.

Neurons rely mainly on oxidative phosphorylation to fuel their high metabolic demands, and any deviation from this norm would lead to neurological disorders [8, 9]. In this current work, we investigated if defective mitochondrial respiration could be the common pathway implicated in both sporadic and familial ALS. To do so, we differentiated patient-derived and isogenic pairs of induced pluripotent stem cells (iPSCs) toward spinal MNs and developed a method to enrich for these MNs for metabolic flux measurements, and, identified reduced mitochondrial respiration and elevated glycolysis as a metabolic hallmark of ALS MNs. We found that the defective mitochondrial respiration was attributed to the hyper-acetylation of mitochondrial proteins caused by reduced Sirtuin-3 (SIRT3) activity. Using nicotinamide (NAM) and small molecule activators to restore SIRT3 activity, we were able to rescue the metabolic defects and also improve ALS MN morphology and survival. Our work demonstrates that hyper-acetylated mitochondrial proteins are hallmarks of both sporadic and familial ALS and elevating SIRT3 activity can be explored as a therapeutic strategy for ALS.

## Results

### MNs generated from ALS patient iPSCs and isogenic ALS knock-in iPSCs exhibit reduced mitochondrial respiration and ATP production

**Fig. 1:**
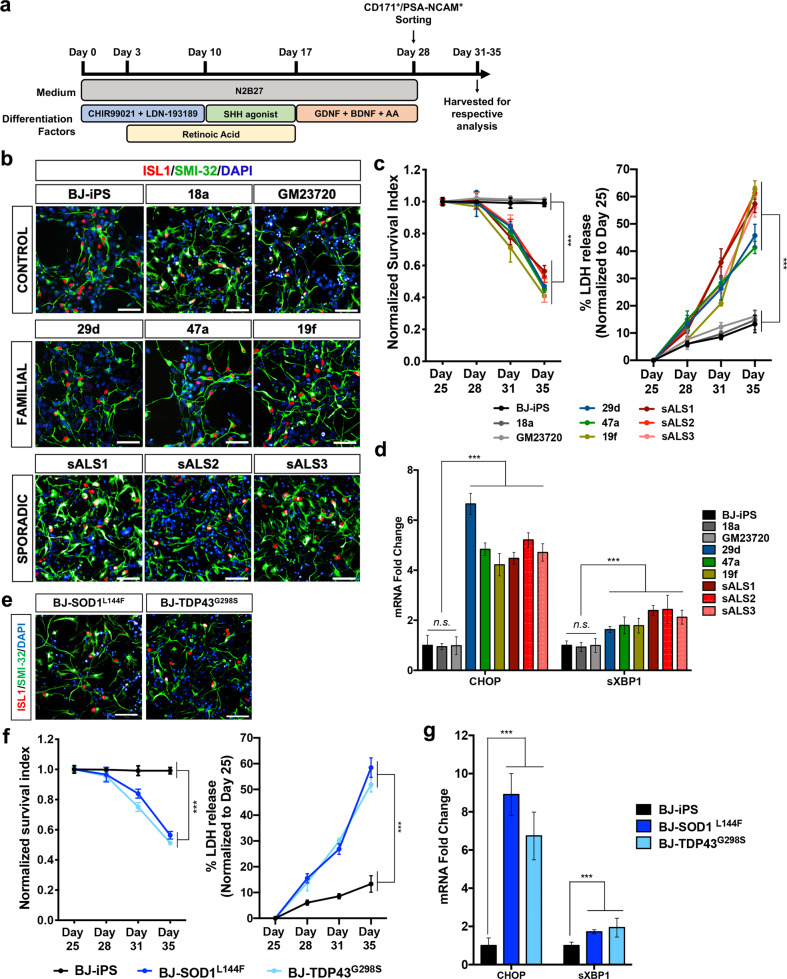
ALS iPSC-derived MNs exhibit diseased phenotypes. **a** Schematic of the MN differentiation protocol. **b** Immunostaining of wild-type (BJ-iPS, 18a, and GM23720), familial ALS (29d, 47a, and 19f), and sporadic ALS (sALS1, sALS2, and sALS3) iPSC-derived cultures at day 28 indicating the derivation of ISL1^+^SMI32^+^ MNs. Cellular nuclei were counterstained with DAPI. Scale bars, 50 μm. **c** Quantification of ISL1^+^ MNs from days 25 to 35 demonstrating that wild-type MNs (BJ-iPS, 18a, and GM23720) remain viable while familial and sporadic ALS MNs show significantly reduced survival over time. Quantification of lactate dehydrogenase (LDH) leakage from days 25 to 35 demonstrating that ALS MNs have significantly higher leaked LDH as compared to WT MNs. **d** qPCR quantification of ER stress transcripts *CHOP* and spliced *XBP1* (*sXBP1*) in MN cultures at day 28. Fold changes are normalized to expression levels of respective mRNA in BJ-iPS. **e** Immunostaining of isogenic ALS (BJ-SOD1^L144F^ and BJ-TDP43^G298S^) iPSC-derived cultures at day 28 indicating the formation of ISL1^+^SMI32^+^ MNs. Cellular nuclei were counterstained with DAPI. Scale bars, 50 μm. **f** Quantification of ISL1^+^ MNs and leaked LDH derived from BJ-SOD1^L144F^ and BJ-TDP43^G298S^ from days 25 to 35 revealed an accelerated death phenotype and increased LDH leakage similar to that of other ALS lines. **g** MN cultures derived from BJ-SOD1^L144F^ and BJ-TDP43^G298S^ show upregulation of *CHOP* and *sXBP1* compared to its isogenic control line BJ-iPS. In (**d**, **g**), gene expression was normalized to ACTINB and HPRT. ***p < 0.001, ns non-significant; one-way ANOVA, Tukey’s multiple comparisons post hoc test.

MNs were differentiated from three healthy iPSC lines: BJ-iPS, 18a, and GM23720, three sporadic ALS lines: sALS1, sALS2, and sALS3, as well as familial ALS iPSCs (harboring the following mutations): 29d (SOD1^L144F^), 49a (TDP43^G298S^), and 19f (C9ORF72 expanded GGGGCC repeats) [10] using established protocols (Fig. 1a). By day 28, ISL1^+^SMI32^+^ MNs were effectively derived from all of these iPSC lines (Fig. 1b and Supplementary Fig. 1a). To investigate accelerated MN death, we measured basal survival of ISL1^+^ MNs and lactate dehydrogenase (LDH) leakage in MN cultures from days 25 to 35. Our results indicate that MN numbers decreased over time, while LDH leakage was elevated in the ALS lines, indicating that ALS MNs displayed an accelerated death phenotype (Fig. 1c). Since elevated ER stress is also a molecular signature of ALS MNs [11], we measured mRNA levels of key genes in the ER stress pathway and found significant upregulation of *CHOP* and spliced XBP1 (*sXBP1*) (Fig. 1d).

To ensure that the ALS-specific phenotypes and molecular profiles are not due to inherent variability between cell lines, we generated isogenic controls where we introduced the SOD1^L144F^ and TDP43^G298S^ mutations into the healthy BJ-iPS line (Fig. 1e and Supplementary Fig. 1b, c). Similar to what was observed with the patient iPSC lines, MNs derived from the isogenic SOD1^L144F^ (BJ-SOD1^L144F^) and TDP43^G298S^ (BJ-TDP43^G298S^) knock-in lines showed decreased MN survival, increased LDH leakage, and elevated expression of ER stress genes *CHOP* and *sXBP1* (Fig. 1f, g).

**Fig. 2:**
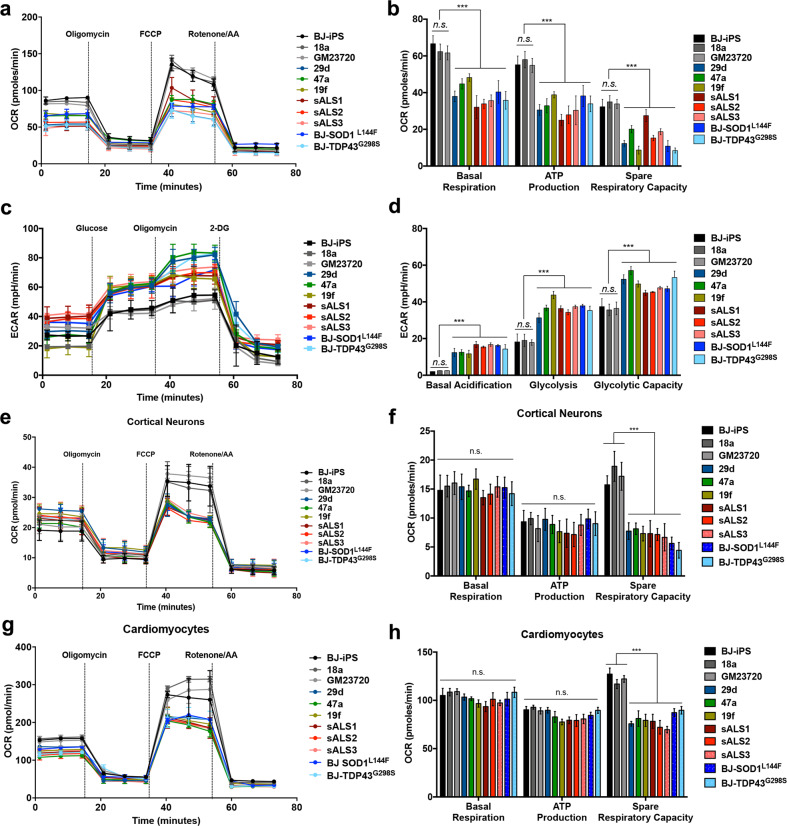
Sporadic and familial ALS MNs show a hypo-oxidative and hyper-glycolytic metabolic profile. **a** Metabolic flux plots of healthy and ALS patient-derived sorted neurons, where oxygen consumption rate (OCR) was measured as a function of time. The MitoStress assay was used to measure bioenergetics parameters, by adding Complex V inhibitor oligomycin, mitochondrial uncoupler FCCP, and Complexes I and III inhibitors rotenone and antimycin A (AA). **b** Basal respiration, ATP production, and spare respiration were calculated for sorted neurons from each of the cell lines and demonstrated reduced mitochondrial respiration in ALS MNs. **c** Metabolic flux plots of healthy and ALS patient-derived sorted neurons, where extracellular acidification rate (ECAR) was measured as a function of time. The Glycolysis stress assay was used to measure bioenergetics parameters, by adding glucose, Complex V inhibitor oligomycin, and hexokinase inhibitor 2-DG. **d** Basal acidification, glycolysis, and glycolytic capacity were calculated for sorted neurons from each of the cell lines and demonstrated elevated glycolysis in ALS MNs. **e** OCR measurements using the MitoStress assay were performed and calculated for cortical neurons derived from healthy, ALS, and diseased isogenic iPSCs at day 28. **f** Metabolic flux analyses were performed using the MitoStress assay. Basal respiration, ATP production, and spare respiration calculated for BJ-iPS, BJ-SOD1^L144F^, and BJ-TDP43^G298S^ cortical neurons reveal no significant changes in basal respiration and ATP production. Likewise, healthy and ALS patient iPSC-derived cortical neurons reveal no significant changes in basal respiration and ATP production. **g** OCR measurements using the MitoStress assay were performed and calculated for cardiomyocytes derived from healthy, ALS, and diseased isogenic iPSCs at day 28. **h** Metabolic flux analyses were performed using the MitoStress assay. Basal respiration, ATP production, and spare respiration calculated for BJ-iPS, BJ-SOD1^L144F^, and BJ-TDP43^G298S^ cardiomyocytes reveal no significant changes in basal respiration and ATP production. Likewise, healthy and ALS patient iPSC-derived cardiomyocytes reveal no significant changes in basal respiration and ATP production. ***p < 0.001, ns non-significant; one-way ANOVA, Tukey’s multiple comparisons post hoc test.

Since oxidative phosphorylation is critical for maintenance of neuronal metabolism and survival, we investigated if mitochondrial respiration in ALS MNs could be compromised. To enrich for MNs in our iPSC-derived cultures, we performed magnetic sorting using a cocktail of PSA-NCAM and CD171 antibodies. Using this sorting strategy, we enriched for ISL1^+^ MNs to ~60% (Supplementary Fig. 2a, b) without the use of AraC, which is sometimes used to deplete neural progenitor cells (NPCs) in the cultures but also induces neuronal death through oxidative stress [12]. To investigate whether ALS MNs exhibit metabolic respiration defects, oxygen consumption rate (OCR) of these sorted neurons was measured as a function of time using an extracellular flux analyzer. We found that both familial and sporadic ALS lines displayed significantly reduced basal respiration, decreased ATP-linked OCR as well as spare respiratory capacity compared to the healthy MNs (Fig. 2a, b). Likewise, MNs derived from BJ-SOD1^L144F^ and BJ-TDP43^G298S^ isogenic iPSCs exhibited reductions in basal respiration (*p* < 0.0001), ATP production (*p* < 0.0001), and spare respiratory capacity (*p* < 0.01), similar to 29d and 47a MNs that also possess the heterozygous L144F mutation in SOD1 and G298S mutation in TDP43, respectively (Fig. 2a, b).

To investigate whether disruptions of mitochondrial respiration would impact MN survival, we treated BJ-iPS MNs with low, nonlethal doses of oligomycin (ATP Synthase inhibitor) or rotenone (Complex I inhibitor) from days 28 to 31. We found that treatment using 2 nM oligomycin resulted in 50% MN death and while rotenone treatment at 2 nM resulted in 25% MN death (Supplementary Fig. 2c). In both conditions, MNs are more vulnerable than the rest of the culture, mimicking the ALS phenotype and suggesting that maintenance of mitochondrial respiration is essential for MN survival.

Since ATP production in ALS MNs is significantly reduced, we reasoned that these neurons would have to turn to other sources of energy to fuel their metabolic needs. One possibility is to derive additional ATP through glycolysis. To investigate if these neurons switch to glycolysis to meet their energy demands, we measured extracellular acidification rate (ECAR) using a metabolic flux analyzer. ECAR measurements revealed that ALS MNs exhibited increased basal acidification, glycolysis, and glycolytic capacity when compared to healthy MNs (Fig. 2c, d), confirming that the ALS MNs have a hyper-glycolytic phenotype. Similarly, this hyper-glycolysis was recapitulated in BJ-SOD1^L144F^ and BJ-TDP43^G298S^ MNs compared to BJ-iPS MNs, revealing that this is an ALS-specific metabolic feature rather than due to variation between cell lines (Fig. 2c, d). To provide further evidence of hyper-glycolysis in ALS MNs, we measured the amount of lactate secreted into culture media as high lactate levels tend to associate with hyper-glycolysis. We found increased lactate levels in culture media derived from ALS MNs (Supplementary Fig. 2d), providing further evidence of a hyper-glycolytic metabolic phenotype in ALS MNs.

To assess the specificity of these metabolic changes in MNs, we measured OCR of day 10 NPCs and found that ALS NPCs revealed no significant changes to the basal respiration and ATP-linked OCR. However, spare respiratory capacity was reduced by approximately half in ALS NPCs (Supplementary Fig. 2e, f). Furthermore, glycolysis profiles of day 10 NPCs do not show significant changes between healthy and ALS cells (Supplementary Fig. 2g, h).

### Other metabolically active cell types derived from ALS iPSCs do not show overt reduction in mitochondrial respiration

Next, we wondered if ALS-causing mutations affect all actively respiring cell types or specifically MNs. To this end, we differentiated all iPSCs toward BRN2^+^SATB2^+^ cortical neurons using a 28-day protocol (Supplementary Fig. 3a–c). These neurons were then replated on 96-well plates for metabolic flux analyses. OCR measurements revealed that basal respiration and ATP production were not significantly different between BJ-SOD1^L144F^ and BJ-TDP43^G298S^ cortical neurons versus the isogenic control (Fig. 2e, f). Likewise, cortical neurons derived from familial and sporadic ALS iPSCs do not show significant differences in basal respiration and ATP production compared to the three healthy controls (Fig. 2e, f). The spare respiratory capacity, which measures the additional energy production by oxidative phosphorylation in response to sudden energy requirements, is more than halved in all the ALS cortical neurons, suggesting that these cells have a normal basal respiration but may be more susceptible to metabolic stress.

To investigate nonneural cell types, cTnT^+^ cardiomyocytes were also derived from all the iPSCs (Supplementary Fig. 3d) and metabolic flux analyses were performed. BJ-SOD1^L144F^ and BJ-TDP43^G298S^ cardiomyocytes were not significantly different from their isogenic healthy control in terms of basal respiration and ATP production, although total respiratory capacity was also reduced (Fig. 2g, h). Likewise, similar results were also obtained for familial and sporadic ALS patient-derived cardiomyocytes (Fig. 2g, h). Taken together, our results revealed that reduced basal mitochondrial respiration and the concomitant elevation of glycolysis are metabolic hallmarks of ALS MNs.

### ALS MN metabolic defects associated with hyper-acetylation of mitochondrial proteins

**Fig. 3:**
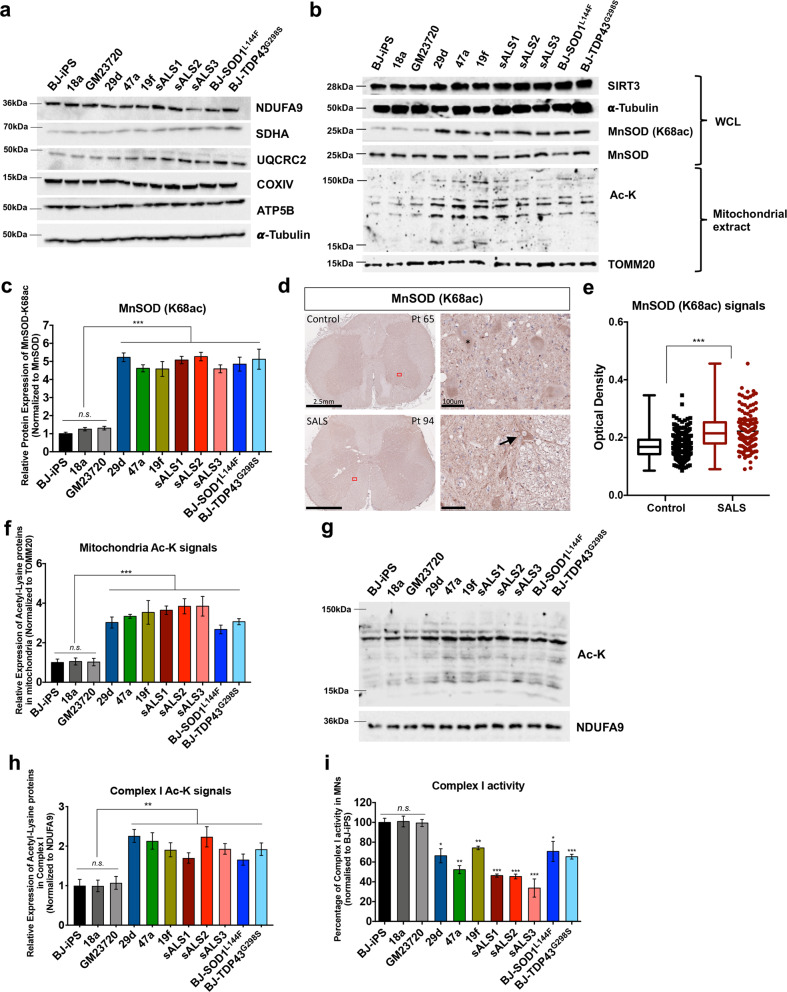
Hyper-acetylation of mitochondrial proteins in familial and sporadic ALS MNs. **a** Western blot analyses at day 28 revealed no significant changes in levels of mitochondrial proteins in both WT and ALS MNs. **b** Western blot analyses of iPSC-derived MNs at day 28 probing for SIRT3, total MnSOD, MnSOD with specific acetylation at lysine 68 (MnSOD K68ac) on whole-cell lysate as well as probing for acetyl-lysine proteins in purified mitochondrial extracts. **c** Quantitative analyses of Western blot bands where MnSOD(K68ac) levels were normalized to total MnSOD levels revealed between 4-fold to 5.5-fold increase in MnSOD(K68ac) in all the ALS iPSC-derived MNs compared to healthy MNs. **d** Immunohistochemistry of control and sporadic ALS patients (SALS) lumbar sections revealed increased MnSOD (K68ac) signals in SALS patients lumbar motor neurons (arrow). Lipofuscin is visible in large, healthy motor neurons, a function of normal cellular aging and unrelated to disease (*). Scale bars, 2.5 mm (left panel) and 100 μm (right panel). **e** Quantification of MnSOD (K68ac) signals in both control (*n* = 380) and SALS (*n* = 216) lumbar motor neurons demonstrates increased MnSOD (K68ac) signals in SALS patients. ***p < 0.001; Two-tailed *t-*test. **f** Densitometric analysis of acetyl-lysine signals normalized to TOMM20 revealed approximately threefold increase in acetylation of mitochondria proteins in all of our ALS iPSC-derived MNs. **g**, **h** Immunoprecipitation of Complex I subunits demonstrated increased acetyl-lysine signals in ALS MNs. **i** Complex I activity in healthy and ALS MNs was measured, which revealed significant decline of between 30 and 80% in the ALS MNs. *p < 0.05, **p < 0.01, ***p < 0.001, ns non-significant; one-way ANOVA, Tukey’s multiple comparisons post hoc test.

Having established that mitochondrial respiration defects are associated with both familial and sporadic ALS, we sought to understand the mechanisms that underlie these defects. We first investigated if the mitochondrial respiration defects in ALS MNs were attributed to reduced expression of mitochondrial proteins. However, western blot analysis of the different subunits of the electron transport chain did not reveal significant changes between healthy and ALS MNs (Fig. 3a).

Mitochondrial respiration is fine-tuned by the acetylation of mitochondrial proteins, and this process is controlled by the mitochondrial deacetylase SIRT3 [13]. Since it has been previously reported that SIRT3 depletion leads to mitochondrial protein hyper-acetylation in muscles, cardiac, and liver tissues [14], we investigated if the loss of SIRT3 resulted in the ALS-specific metabolic defects we observed. However, both western blot and quantitative-PCR analysis did not reveal significant changes in SIRT3 levels between healthy and ALS MNs (Fig. 3b and Supplementary Fig. 4a, b). We postulate that SIRT3 activity could be affected even though expression levels were unchanged. Therefore, to determine SIRT3 activity, we measured relative acetylation of lysine 68 on MnSOD (MnSOD K68ac), one of the best-characterized SIRT3 target [15, 16]. Indeed, western blot analyses revealed much higher MnSOD K68ac in all of our ALS MN cultures, including the sporadic ALS MNs and isogenic MNs, compared to healthy controls, suggesting reduced SIRT3 activity (Fig. 3b, c). Human postmortem lumbar spinal cord sections were also analyzed by immunohistochemistry, which confirmed higher MnSOD-K68ac signals in alpha MNs of lumbar spinal cord in sporadic ALS patients compared with non-ALS controls (Fig. 3d, e). Since SIRT3 has multiple targets in the mitochondria, it is expected that loss of SIRT3 activity would impact global mitochondrial acetylation. To confirm this, we harvested mitochondrial extracts from all our iPSC-derived MNs and performed immunoblotting of acetylated proteins using an acetyl-lysine antibody. Western blot analysis revealed significantly higher intensities of acetylated proteins in all of the ALS lines compared to the healthy controls, concurring with the MnSOD-K68ac assay (Fig. 3b, f). Previous SIRT3 interactome studies have revealed several Complex I subunits among its downstream targets [17]. To confirm if Complex I subunits were hyper-acetylated in ALS neurons, co-immunoprecipitation using a Complex I Immunocapture antibody was performed, followed by immunoblotting of acetylated proteins. This revealed significantly higher acetylation of Complex I subunits in all the ALS MNs compared to healthy MNs (Fig. 3g, h). Reduced Complex I activity were also observed in the ALS MNs compared to healthy controls (Fig. 3i). Taken together, these data indicate that hyper-acetylation of Complex I subunits may lead to decreased activity, providing an explanation for the lower basal mitochondrial respiration observed in these ALS MNs (Fig. 2).

### Loss of SIRT3 function mimics ALS phenotypes

**Fig. 4:**
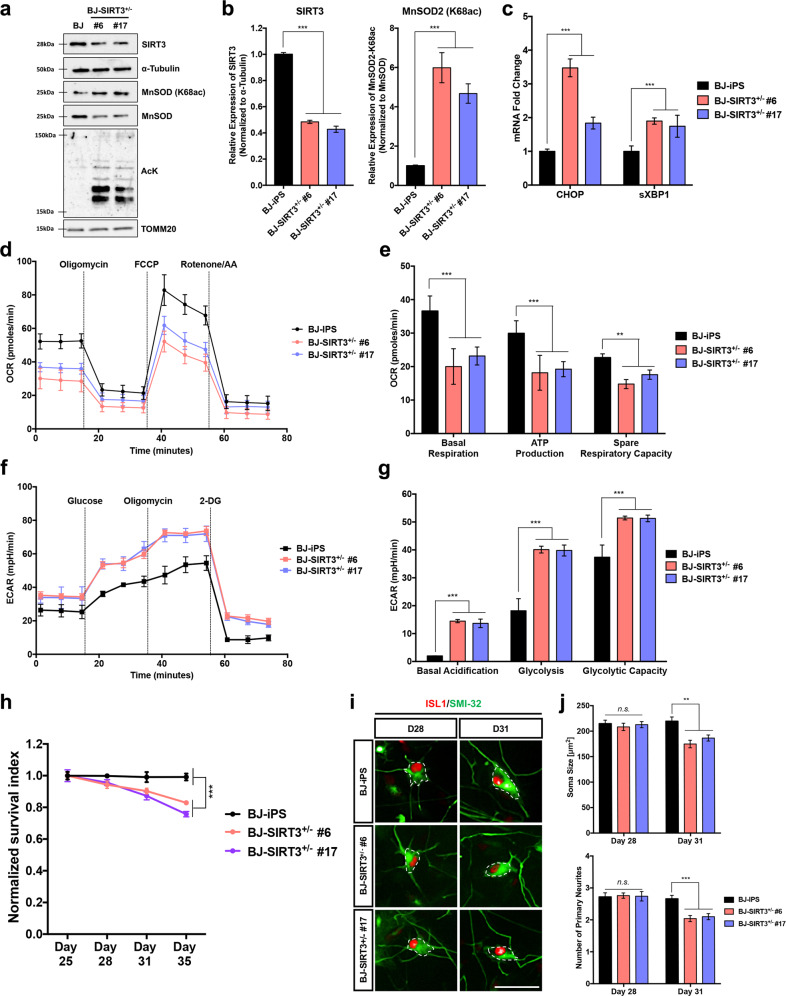
SIRT3 deficiency in MNs results in ALS-like phenotypes. **a** Western blot analysis of day 28 MNs derived from BJ-iPS and two isogenic SIRT3^+/−^ (#6 and #17) clones confirmed reduction in SIRT3 protein, along with increased MnSOD (K68ac) and increased acetylation of mitochondrial proteins. **b** Densitometric analyses of western blot bands reveal 50% decrease in SIRT3 protein levels and increased MnSOD (K68ac) in both SIRT3^+/−^ #6 and #17 versus healthy MNs. **c** qPCR measurements of *CHOP* and *sXBP1* show significant upregulation of both ER stress transcripts in SIRT3^+/−^ #6 and #17 relative to the isogenic BJ-iPS control. **d** Measurements of OCR using the MitoStress assay of day 28 MNs differentiated from BJ-iPS (shown in black), SIRT3^+/−^ #6 (pink), and #17 (violet). **e** Measurements of basal respiration, ATP production, and spare respiration of day 28 MNs differentiated from BJ-iPS (shown in black), SIRT3^+/−^ #6 (pink), and #17 (violet). **f** Measurements of ECAR using the Glycolysis Stress assay of day 28 MNs differentiated from BJ-iPS (shown in black), SIRT3^+/−^ #6 (pink), and #17 (violet). **g** Measurements of basal acidification, glycolysis, and glycolytic capacity of day 28 MNs differentiated from BJ-iPS (shown in black), SIRT3^+/−^ #6 (pink), and #17 (violet). **h** Quantification of ISL1^+^ MNs derived from both BJ-SIRT3^+/−^ clones from days 25 to 35 revealed a progressive death phenotype as compared to its healthy control (BJ-iPS). **i** Representative images of ISL1^+^SMI32^+^ MNs derived from BJ-iPS, BJ-SIRT3^+/−^ #6 and #17 iPSCs, showing cell body sizes (outlined in white dotted lines) from MNs at day 28, and at day 31. Scale bars, 50 μm. **j** Quantification of mean cell body size and number of primary neurites of both BJ-SIRT3^+/−^ clones reveal deteriorating neuronal health from days 28 to 31. **p < 0.01, ***p < 0.001, ns non-significant; one-way ANOVA, Tukey’s multiple comparisons post hoc test.

To assess the function of SIRT3 in regulating mitochondrial bioenergetics, we ablated SIRT3 expression to study its effect on MN survival and function. Using the CRISPR/Cas9 approach, we generated multiple isogenic SIRT3 haploinsufficient (SIRT3^+/−^) iPSC lines (Supplementary Fig. 1d). We randomly selected SIRT3^+/−^ clones #6 and #17 and showed that both clones differentiated into MNs with approximately the same efficiencies as the isogenic control BJ-iPS (Supplementary Fig. 1e). Western blot analysis confirmed that both clones showed 50% reduction of SIRT3 protein. MnSOD-K68ac levels were also significantly higher in BJ-SIRT3^+/−^ #6 and #17, indicating that SIRT3 is important in regulating mitochondrial protein acetylation (Fig. 4a, b). Furthermore, purified mitochondrial extracts also showed that clones #6 and #17 had more acetyl-lysine residues (Fig. 4a), consistent with the hyper-acetylated mitochondrial profiles seen in the ALS iPSC-derived MNs (Fig. 3a). A constellation of in vitro ALS phenotypes has now been reported, which includes accelerated MN death, elevated ER stress signaling, smaller cell bodies [11, 18], and a hypo-oxidative/hyper-glycolytic metabolic profile (Fig. 2). Therefore, we sought to determine if loss of SIRT3 could induce ALS phenotypes in vitro. Measurements of ER stress transcripts in the day 28 MNs from both SIRT3^+/−^ clones revealed significant upregulation of *CHOP* and *sXBP1* mRNAs (Fig. 4c), similar to that seen in all the ALS MNs we tested (Fig. 1d). Metabolic flux measurements confirmed that MNs derived from both SIRT3^+/−^ clones exhibited reduced mitochondrial respiration (Fig. 4d, e) and simultaneous elevated glycolysis (Fig. 4f, g), similar to the profile seen in ALS MNs (Fig. 2a–d). Phenotypically, MNs derived from both SIRT3^+/−^ clones had reduced survival (Fig. 4h) and significantly reduced soma sizes and primary neurites at day 31 (Fig. 4i, j). Given that SIRT3^+/−^ MNs display ALS-like phenotypes, this suggests that partial loss of SIRT3 activity contributes to ALS pathogenesis.

Additionally, we performed small interfering RNA (siRNA)-mediated knockdown to transiently deplete SIRT3 levels in healthy BJ-iPS MNs at day 25. At day 28, we confirmed 70% knockdown at both mRNA and protein levels, together with increased MnSOD K68ac, indicative of decreased SIRT3 activity (Supplementary Fig. 4c, d). Analyses of these neurons at day 28 also revealed reduced OCR parameters (Supplementary Fig. 4e). Taken together, these results suggest that SIRT3 is important in maintaining mitochondrial bioenergetics in MNs and depletion of SIRT3 leads to ALS-like phenotypes.

### SIRT3 activation improves metabolic defects in ALS MNs

**Fig. 5:**
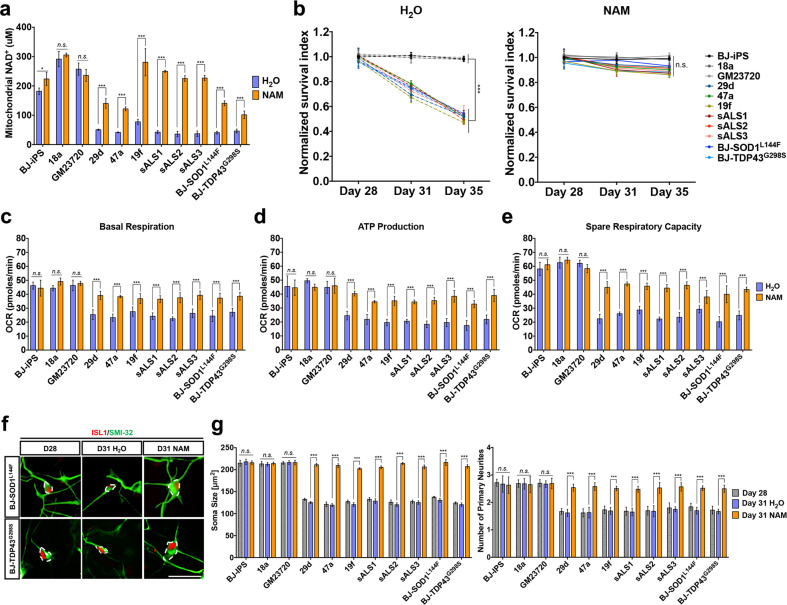
NAM supplementation reverses mitochondrial respiration defect and improves neuronal morphology in ALS MNs. **a** Measurements of mitochondrial NAD^+^ levels in healthy and ALS MNs after NAM supplementation revealed increased mitochondrial NAD^+^ availability in ALS MNs. **b** WT and ALS iPSC-derived MNs were treated with either H_2_O or NAM from days 28 to 35. Number of ISL1^+^ MNs were quantified and normalized to number of ISL1^+^ MNs in respective cell lines at day 28. NAM supplementation prevents MN death in ALS MNs. One-way ANOVA with Tukey’s multiple comparisons post hoc test has been performed to analyze survival of wild-type and ALS MNs on day 35. **c**–**e** Measurements of basal respiration, ATP production, and spare respiration, respectively, in healthy and ALS MNs treated with water as a control (purple) or 0.5 mM NAM (orange). **f** Representative images of ISL1^+^SMI32^+^ MNs of BJ-SOD1^L144F^ and BJ-TDP43^G298S^ illustrating NAM supplementation promotes healthier neuronal morphologies. MN cell body sizes are outlined by white dotted lines. Scale bars, 50 μm. **g** Measurement of soma size and primary neurites shows overall improvement in neuronal morphology in NAM supplemented ALS MNs. ***p < 0.001, ns non-significant; two-tailed *t-*test.

Since SIRT3 is a NAD^+^-dependent deacetylase, one possibility for low SIRT3 activity is reduced mitochondrial NAD^+^ in ALS MNs. To investigate this, we measured NAD^+^ levels in mitochondria isolated from iPSC-derived MNs and found 60–80% reduction in NAD^+^ levels in the mitochondria of sporadic, familial, and isogenic ALS MNs when compared to healthy controls (Fig. 5a). To increase mitochondrial NAD^+^, 0.5 mM NAM was used, resulting in significantly elevated mitochondrial NAD^+^ in ALS MNs (Fig. 5a). We found that NAM treatment promoted ALS MN survival (Fig. 5b) and improved basal mitochondrial respiration, ATP production as well as spare respiratory capacity (Fig. 5d, e), reversing ALS phenotypes. Moreover, addition of exogenous NAM to cell culture media was able to promote healthier neuronal morphologies similar to that of wild-type MNs (Fig. 5f, g). It is worth pointing out that NAM supplementation did not elevate mitochondrial NAD^+^ levels and had no significant effects on mitochondrial respiration of healthy MNs, indicating targeted rescue of ALS MNs.

Supporting the view that mitochondrial NAD^+^ was the limiting factor, we demonstrated that overexpression of SIRT3 using an inducible lentiviral system did not result in significant changes in MnSOD-K68ac signals in ALS MNs (Supplementary Fig. 4f). Likewise, SIRT3 overexpression alone had no significant effects on mitochondrial respiration parameters in the ALS MNs (Supplementary Fig. 4g–i), suggesting that mitochondrial NAD^+^ levels and SIRT3 activity are limiting in the ALS MNs. Therefore, it is not surprising that simply over-expressing SIRT3 without the addition of NAM or other NAD^+^ precursors would have no significant effects on mitochondrial respiration in ALS MNs.

### A small molecule activator of SIRT3, but not riluzole or edaravone, improves mitochondrial bioenergetics in ALS MNs

**Fig. 6:**
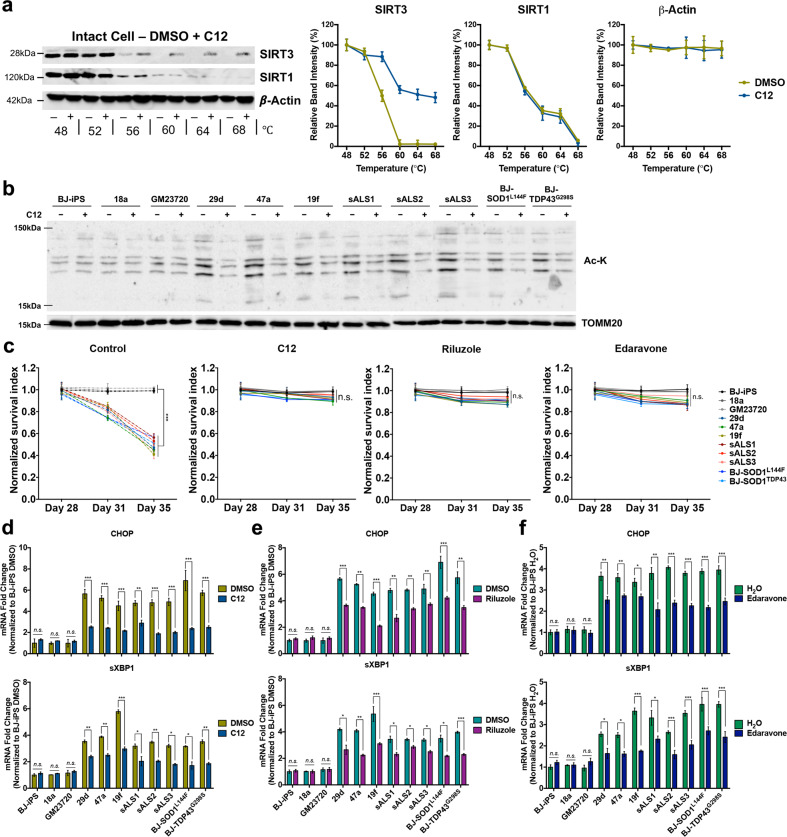
A small molecule activator of SIRT3, but not riluzole or edaravone, improves neuronal morphologies in ALS MNs. **a** Representative western blot and CESTA melt curves in intact cell for SIRT3 target with C12 (at 20 μM). **b** Western blot analyses at day 31 revealed reduction in mitochondria Ac-K signals in ALS MNs upon C12 treatment. **c** WT and ALS iPSC-derived MNs were treated with either DMSO/H_2_O or C12/riluzole/edaravone from days 28 to 35. Number of ISL1^+^ MNs were quantified and normalized to number of ISL1^+^ MNs in respective cell lines at day 28. C12, riluzole, and edaravone treatment prevents MN death in ALS MNs. One-way ANOVA with Tukey’s multiple comparisons post hoc test has been performed to analyze survival of wild-type and ALS MNs on day 35. **d**–**f** qPCR quantification of ER stress transcripts CHOP and spliced XBP1 (sXBP1) in MN cultures at day 31 treated with controls or C12/riluzole/edaravone. Fold changes are normalized to expression levels of respective mRNA in BJ-iPS MNs treated with DMSO or water. Gene expression was normalized to ACTINB and HPRT. ***p < 0.001, ns non-significant; two-tailed *t-*test.

A small number of SIRT3 activators have been identified, including a specific SIRT3 agonist previously identified as 7-hydroxy-3-(4′-methoxyphenyl) coumarin or C12 that changes the conformation of SIRT3 active site with high affinity and promotes deacetylation of downstream targets [16]. To evaluate the specificity of C12 binding to SIRT3, we performed cellular thermal shift assay (CETSA) [19] and confirmed that C12 promotes thermal stability of SIRT3, but not another abundantly expressed sirtuin, SIRT1 (Fig. 6a). Next, a dose response assay for C12 was performed by treating MNs derived from one of the ALS lines (BJ-SOD1^L144F^) with increasing doses of C12. Western blot analysis showed reduction of MnSOD-K68ac signals in a dose-dependent manner, while SIRT3 and total MnSOD protein levels remain unchanged (Supplementary Fig. 5a, b). We noted that treatment with 10 μM C12 was cytotoxic (Supplementary Fig. 5c, d), and subsequent experiments were therefore conducted using 5 μM C12. By treating the panel of ALS MNs with 5 μM C12, we demonstrated that mitochondrial acetyl-lysine signals were significantly reduced in all the ALS cultures (Fig. 6b), confirming that C12 promotes mitochondrial deacetylation.

We next compared the effects of C12 vis-à-vis the FDA-approved treatments for ALS, namely, riluzole and edaravone. Riluzole functions by inhibiting glutamate release and blocking glutamatergic transmission, thereby reducing excitotoxicity, while edaravone works as a reactive oxygen species (ROS) scavenger. However, it is currently not known whether riluzole and edaravone would regulate mitochondrial respiration. To this end, we treated the iPSC-derived MNs with C12, riluzole, edaravone, and their respective controls from days 28 to 35. First, we found that all three compounds promoted ALS MN survival (Fig. 6c) and significantly reduced expression of ER stress transcripts *CHOP* and *sXBP1* in all the ALS MNs at day 31 (Fig. 6d–f).

**Fig. 7:**
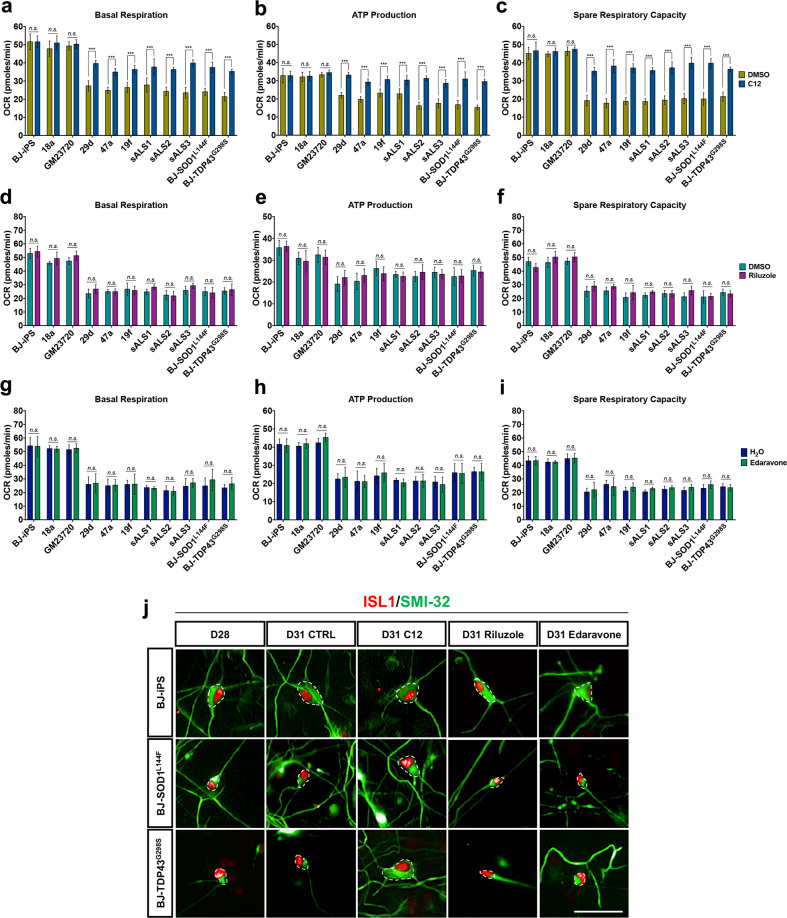
A small molecule activator of SIRT3, but not riluzole or edaravone, reverses metabolic defects in ALS MNs. **a**–**c** Measurements of basal respiration, ATP production, and spare respiration, respectively, in healthy and ALS MNs treated with DMSO (green) and C12 (blue). Of note, C12 treatment rescued ATP production back to healthy levels. **d**–**f** Measurements of basal respiration, ATP production, and spare respiration, respectively, in healthy and ALS MNs treated with DMSO (turquoise) and riluzole (purple). Of note, riluzole treatment is not able to revert ALS MNs mitochondrial bioenergetics defects. **g**–**i** Measurements of basal respiration, ATP production, and spare respiration, respectively, in healthy and ALS MNs treated with water (dark blue) and edaravone (dark green). Of note, edaravone treatment is not able to revert ALS MNs mitochondrial bioenergetics defects. **j** Representative images of ISL1^+^SMI32^+^ MNs of BJ-iPS, BJ-SOD1^L144F^, and BJ-TDP43^G298S^ illustrate only C12 treatment promotes healthier neuronal morphologies, showing cell body sizes (outlined in white dotted lines) from MNs at day 28, and at day 31. Scale bars, 50 μm. ***p < 0.001, ns non-significant; two-tailed *t-*test.

Metabolic flux analyses at day 31 revealed that C12 promoted mitochondrial respiration (Fig. 7a–c), reduced glycolysis (Supplementary Fig. 5e–g), and increased mitochondrial Complex I activity (Supplementary Fig. 5h) in ALS MNs. Additionally, C12 treatment led to a reduction in mitochondrial ROS levels (Supplementary Fig. 5i), suggesting that SIRT3 activation also promotes the activation of antioxidant defenses in ALS MNs. However, metabolic flux analyses revealed that riluzole and edaravone treatment did not show any significant changes in the metabolic profiles of ALS MNs (Fig. 7d–i), indicating that treatment with either riluzole or edaravone did not improve mitochondrial bioenergetics. C12 treatment also improved neuronal morphologies, resulting in enlarged soma sizes and increased number of primary neurites similar to healthy MNs (Fig. 7j and Supplementary Fig. 6a–c). However, treatment with either riluzole or edaravone did not improve neuronal morphologies (Fig. 7j and Supplementary Fig. 6d–g). Collectively, these data suggest that SIRT3 activation is effective in promoting MN survival and correcting the hyper-glycolytic and hypo-oxidative metabolic phenotype in ALS MNs.

Notably, treatment of BJ-SIRT3^+/−^ #6 and #17 with C12 did not promote survival or improved neuronal morphology (Supplementary Fig. 7a, b). C12 treatment on both SIRT3-deficient clones also did not improve mitochondrial respiration (Supplementary Fig. 7c), providing additional evidence that C12 works through promoting SIRT3 activity rather than via nonspecific off-target effects. In a complementary experiment, cellular NAD^+^ levels were reduced using the NAMPT inhibitor FK866 [20]. NAMPT is a key enzyme in the biosynthesis of NAD^+^, and FK866 treatment in healthy BJ-iPS MNs resulted in reduced MN survival, which was rescued by addition of C12 (Supplementary Fig. 7d). This suggests that SIRT3 activation is important for MN survival under conditions where NAD^+^ levels are limiting.

## Discussion

ALS is a heterogeneous MN disease where it is classically divided into “sporadic” form when there is no family history of ALS or dementia, and “familial” form when there is. However, the distinction between these forms has been quite porous because the same genes which are involved in familial ALS have also been found in apparently sporadic populations [21]. Genes in the same pathway may not be found yet, and a form may appear to be sporadic simply because of de novo mutations, epigenetic mutations, small family lines, or contributions of more than one gene (oligogenic contributions) [21]. Yet all ALS patients have similar clinical manifestations, suggesting the possibility of a converging pathogenic pathway independent of the various genetic mutations known to cause ALS.

In this study, we identified a metabolic hallmark of both sporadic and familial ALS MNs that is characterized by hypo-oxidation and hyper-glycolysis. Previous reports concur with our findings: central nervous system acidosis was reported in SOD1^G93A^ mice [2] while we show increased basal acidification and lactate release from various sporadic and familial ALS MNs. Reductions in cellular respiration were also observed in the postmortem spinal cord of sporadic ALS patients [22, 23]. It is likely that hyper-glycolysis was a compensatory mechanism to overcome the lack of ATP generated through oxidative phosphorylation in ALS MNs, because restoration of mitochondrial respiration through NAM and C12 treatment corrected the hyper-glycolytic metabolic profile.

One of our key findings was that reduced mitochondrial respiration and hyper-acetylated mitochondrial proteins are molecular hallmarks of ALS MNs. MNs derived from familial and sporadic patient iPSCs, as well as isogenic iPSC lines with SOD1^L144F^ and TDP43^G298S^ mutations demonstrate a consistent increase in acetylated mitochondrial proteins, including MnSOD K68ac, a well characterized target of SIRT3. Postmortem analyses of ALS spinal cords further corroborate this finding. This molecular defect was accompanied by a resultant defect in mitochondrial respiration, which was reversed by SIRT3 activation.

In addition to using patient-derived iPSCs, isogenic cell lines were made to confirm our findings that loss of SIRT3 activity was responsible for the mitochondrial metabolic defects, and the constellation of ALS-like phenotypes. We note that we were unable to obtain SIRT3^−/−^ iPSCs in this study, while mice with complete Sirt3-knockout develop normally and are not embryonic lethal, although neuronal survival and function appear to be affected [24]. Nevertheless, our results confirmed that partial loss of SIRT3 was sufficient to cause ALS phenotypes in iPSC-derived MNs.

Apart from reversing the metabolic defects in ALS MNs, SIRT3 activation also rescued other in vitro ALS phenotypes. This suggests that early ALS neuronal deficits are reversible and treatable and that the SIRT3 is a crucial upstream target regulating MN function and integrity. In support of our data, it has also been reported that Sirt3^−/−^ mouse cortical neurons are particularly vulnerable to excitatory, oxidative, and metabolic stress [25]. Our data support recently published work that NAD^+^ levels in ALS patients are reduced compared to the healthy population [26], and confirm that supplementation of NAD^+^ precursors may benefit ALS.

In mice, knockout of intracellular nicotinamide phosphoribosyltransferase (iNAMPT) in projection neurons leads to an MN degeneration phenotype mimicking ALS [27]. iNAMPT functions as the rate-limiting enzyme of the mammalian NAD^+^ biosynthesis salvage pathway, and depletion of iNAMPT in MNs also led to reduced NAD^+^ levels and increased mitochondrial protein acetylation. While multiple studies have shown that low NAD^+^ levels are associated with ALS [26–28], the exact reasons for NAD^+^ decline remain debatable. The balance between NAD^+^ biosynthesis and NAD^+^ degradation determines cellular NAD^+^ bioavailability. In ALS human spinal cords, it has been shown that NAMPT and NMNAT2 expression levels are reduced [28, 29]. On the other hand, the NADase CD38 has been shown to upregulate with advanced age and is responsible for age-dependent NAD^+^ decline in an SIRT3-dependent manner [28, 29]. Therefore, our current study and these reports highlighted above suggest that ALS is a neurodegenerative disease characterized by systemic NAD^+^ deficiency that can benefit from NAD^+^ supplementation and/or SIRT3 activation.

Of interest to the field, we also found that riluzole and edaravone, the two FDA-approved drugs for ALS treatment, were ineffective in reversing the mitochondrial metabolic defect that is signature of ALS MNs. This may explain the limited benefits of the drugs—riluzole was found to be efficacious among bulbar-onset ALS patients and not in subjects with limb-onset ALS, which constitutes most of the ALS patients [30]. On average, riluzole extends lifespan by 3 months, while edaravone showed efficacy only in a small subset of ALS patients [31].

In conclusion, our study using patient-derived and isogenic iPSCs reveals that reduced mitochondrial respiration and elevated glycolysis are metabolic hallmarks of ALS MNs. We have also established that activation of mitochondrial SIRT3 is a target for reversing disease phenotypes for both sporadic and familial ALS. Finally, our data confirm that NAM supplementation, as well as a small molecule SIRT3 agonist, reverses several in vitro ALS phenotypes and has therapeutic potential for development into an effective treatment.

## Methods

### Culture of hiPSC in iPS-Brew hPSC medium

All hiPSC lines were cultured feeder-free on Matrigel-coated dishes with iPS-Brew XF according to product’s instructions. Routine passaging using ReLeSR (Stem Cell Technologies) was performed once every 6–7 days. The iPSC lines, and their mutations, used in this study are listed in Supplementary Table S1.

### Directed differentiation toward motor neurons

Pluripotent stem cells were differentiated toward the spinal MN fate following established protocols described previously [32]. Briefly, we first neuralized the human iPSC by activating Wnt pathways with CHIR99021 treatment (4.25 μM) while blocking bone morphogenic protein (BMP) signaling by LDN-193189 treatment (0.5 μM) at the same time. At day 3, retinoic acid (1 μM) and purmorphamine (1 μM) were used to caudalize and ventralize the cultures, respectively. Neurotrophic factors, BDNF (10 ng/ml) and GDNF (10 ng/ml), were added to the neuronal cultures at day 17 to promote neuronal maturation into MNs. N2B27 media (50% DMEM/F12, 50% Neuro medium, 1% L-Glutamax, 1% MEM Non-Essential Amino Acids supplemented with 1% N2 supplement, and 2% B27 supplement) was used throughout the MN differentiation.

### Directed differentiation toward cortical neurons

Pluripotent stem cells were differentiated toward the cortical neuron fate following established protocols described previously [33] with slight modifications. We first neuralized the human iPSC with SB431542 treatment (8 μM) to inhibit TGF-β pathway while blocking BMP signaling with LDN-193189 treatment (0.5 μM) at the same time. At day 14, cortical neural progenitors were further differentiated with the addition of DAPT (2.5 μM) for another 7 days. Neurotrophic factors, BDNF (10 ng/ml) and GDNF (10 ng/ml), were added to the neuronal cultures at day 21 to promote neuronal maturation into cortical neurons. N2B27 media lacking vitamin A (50% DMEM/F12, 50% Neuro medium, 1% L-Glutamax, 1% MEM Non-Essential Amino Acids supplemented with 1% N2 supplement, and 2% B27 supplement without vitamin A) was used throughout the cortical neuron differentiation.

### Directed differentiation toward cardiomyocytes

Pluripotent stem cells were differentiated toward the cardiomyocytes fate following established protocols described previously [34]. Briefly, we first directed the human iPSC to mesendoderm by activating Wnt pathways with CHIR99021 treatment (12 μM) for 1 day in RPMI/B27 lacking insulin. To direct these mesendoderm progenitor cells to a cardiac fate, IWP2 (5 μM) was added to the cultures for 2 days in RPMI/B27 lacking insulin on day 3. At day 7, cardiac mesoderm cells were cultured in RPMI/B27 with insulin for development of functional contracting cardiomyocytes.

### Cas9-mediated knockout/knockin in BJ-iPS line

Guide RNAs (gRNAs) were designed using Feng Zhang lab’s guide design tool at crispr.mit.edu before it shut down: gRNAs were cloned into a Cas9-containing plasmid PX458 or PX459 and transfected into 293T cells using Lipofectamine 2000 Transfection Reagent for surveyor nuclease assay. Verified gRNAs and ssODNs (Supplementary Table S2) were transfected into BJ-iPS hiPSCs using Lipofectamine Stem Transfection Reagent. Two days after transfection, cultures were sorted for GFP^+^ cells or selected with 1 µM puromycin. Single cells were then plated out and allowed to expand before screening. gDNA of the colonies were collected and subjected to PCR amplification before sanger sequencing (Applied Biosystems 3730xl).

### Motor neuron survival assay

MN cultures were treated with AraC on day 23 and plated at 75,000 cells per well of a 96-well plate on day 24. Cultures were then fixed with 4% PFA on days 25, 28, 31, and 35, respectively, for quantification of MN survival. Fixed cultures were stained with the MN marker ISL1, and cellular nuclei were counterstained with DAPI before imaging on the high content microscope Phenix (Perkin Elmer). Percentage of ISL1^+^ cells was normalized to total nuclei number. To calculate normalized survival index, the percentage of ISL1^+^/DAPI at day 25 was set as 1.0, and MN survival on later days was, in turn, normalized to day 25 cultures. Biological triplicates were performed with a minimum of five technical replicates each.

### Magnetic microbead sorting of neurons

After dissociating with Accutase, cells were blocked with solution containing phosphate-buffered saline, 0.5% bovine serum albumin (BSA) and 2 mM EDTA. The cells were then incubated with CD171-APC antibody (Miltenyi Biotec) and PSA-NCAM-APC antibody (Miltenyi Biotec) for 10 min at 4 °C. After washing, the cells were then incubated with anti-APC microbeads for 15 min at 4 °C. The cells were then washed twice and filtered prior to loading into the separation column (LS column) that was attached to a magnetic stand (all from Miltenyi Biotec). After three rounds of washing, the column was removed from the magnetic stand and labeled cells were eluted in culture media for replating.

### Metabolic flux analyses using Seahorse XFe96 Analyzer

Mitochondrial OCR and ECAR were measured using a XFe96 Seahorse Biosciences Extracellular Flux Analyzer (Agilent Technologies). Purified MNs or NPCs were plated onto a Matrigel pre-coated Seahorse 96-well plate at 125,000 neurons per well 24 h prior to the assay. Culture media were changed with 175 µl of fresh Seahorse DMEM basal medium 45 min prior to the assay. Seahorse analyzer injection ports were filled with 1 µM oligomycin, 1 µM carbonyl cyanide-4 (trifluoromethoxy) phenylhydrazone (FCCP), or 0.5 µM each of rotenone and antimycin A for OCR. Briefly, oligomycin was first added to the cells to inhibit ATP synthase, which affects the electron flow through the ETC, and a decreased in OCR is linked to cellular ATP production. Then, FCCP, an uncoupling agent, is added to the cells where it collapses the proton gradient and disrupting mitochondrial membrane potential. The electron flow through the ETC becomes uninhibited, and OCR will reach its maximum. The FCCP-stimulated OCR can be used to calculate spare respiratory capacity (difference between maximal OCR and basal OCR), a measurement on the ability for the cell to respond to increased energy demand or under stress. The last modulator added to the cell is a mixture of rotenone and antimycin A, which are Complexes I and II inhibitors, respectively. The mixture shuts down mitochondrial respiration completely, and non-mitochondrial respiration can be calculated. Biological triplicates were performed with a minimum of ten technical replicates each.

ECAR measures the extracellular acid produced by cells, where lactic acid and pyruvic acid are produced by glycolysis and carbon dioxide is produced during the tricarboxylic acid cycle. Briefly, basal acidification, contributed by pyruvic acid, lactic acid, and carbonic acid, is measured at the start of the assay. Upon addition of 10 mM glucose, glycolysis is initiated and the resultant increase in acidification is contributed by pyruvic acid and lactic acid. Next, oligomycin treatment at 1 µM blocks ATP production from mitochondrial respiration and allows the glycolytic capacity of the MNs to be established. Lastly, 50 mM 2-deoxy-glucose (2-DG) acts as a glycolysis inhibitor, and its addition allows for the measurement of non-glycolytic acidification.

Glycolytic capacity was calculated as the difference between ECAR following the injection of 1 μm oligomycin, and the basal ECAR reading. Glycolytic reserve was calculated as the difference in ECAR between glucose and oligomycin injections. Biological triplicates were performed with a minimum of ten technical replicates each.

### Small molecule treatments in MN cultures

Riluzole was reconstituted in DMSO and was used at a final concentration of 5 μM based on previous work [35], while edaravone was dissolved in water and used at a final concentration of 100 μM [36]. NAM and C12 were reconstituted in water and DMSO, respectively, and diluted in media at the desired concentrations of 0.5 mM [37] and 5 μM, respectively. Oligomycin and rotenone were reconstituted in DMSO and diluted in media at the desired concentration of 2 nM. FK866 was reconstituted in DMSO and was used at a final concentration of 1 nM. MNs at day 27 were plated at 75,000 cells per well of a 96-well plate. Treatment with the respective small molecule began at day 28, for a total of 3 or 7 days. Biological triplicates were performed with a minimum of five technical replicates each.

### RNA interference in MN cultures

MN cultures at day 27 were dissociated with Accutase and seeded at 2 million cells per well in a six-well plate. At day 28, non-targeting siRNA or siRNAs were individually complexed with Lipofectamine RNAiMAX following manufacturer’s instructions and added to the MN cultures. For each well, 10 pmol of siRNAs and 8 μl of Lipofectamine RNAiMAX were used. Cells were either harvested for RNA and protein analyses, fixed for immunostaining or metabolic flux analysis were performed 3 days after siRNA transfection.

### RNA extraction and RT-qPCR

Cells were harvested in Trizol reagent for RNA extraction following manufacturer’s instructions. Purified RNA was converted to cDNA using the High-Capacity cDNA Reverse Transcription kit, and quantitative PCR (qPCR) was performed on the QuantStudio 5 Real-Time PCR System using PowerUp™ SYBR™ Green Master Mix (all from Applied Biosystems). Gene expression levels were normalized to HPRT and ACTB expression unless otherwise stated. Primers used are listed in Supplementary Table S3.

### SDS-PAGE and western blot

Protein lysates were resolved in 12% SDS-PAGE gels or 4–20% precast gels in Tris-Glycine-SDS buffer. Proteins were then transferred to a nitrocellulose membrane and blocked with 5% milk in TBST buffer. Primary antibodies were diluted in 5% milk and incubated with the membranes overnight at 4 °C. Primary antibodies used are listed in Supplementary Table S4. Membranes were washed thrice in TBST buffer. The corresponding horseradish peroxidase secondary antibodies (Life Technologies) were then diluted 1:5000 in 5% milk and incubated at room temperature for 90 min. Blots were washed thrice before exposing to ECL for imaging.

### Cellular thermal shift assay (CETSA)

CETSA was performed as previously described [19] and HEK293T cells were used to carry out the experiment. Briefly, 30 million cells were exposed to a final concentration of 20 μM C12 or DMSO for 1 h on low-attachment plates. After the incubation, the cells were harvested, washed, pellet down, and resuspended in 1 mL of PBS. Equal amounts of cell suspensions were aliquoted into Eppendorf tubes. The cell suspensions were then heated (48–68 °C) and lysed using 2 cycles of freeze-thawing. The soluble fractions were isolated and analyzed by western blot analysis as described above.

### Mitochondria isolation

Mitochondria from MN cultures were isolated using the MACS technology. MNs culture was first dissociated using Accutase and washed twice with PBS before resuspending in ice cold Lysis buffer. The cells were then homogenized with a dounce homogenizer (Pestle B) with 15 strokes. The homogenate was diluted with 1X separation buffer based on manufacturer’s instructions. Anti-TOM22 were added to magnetically label the mitochondria before incubating at 4 °C for 1 h. The cells were then washed twice and filtered prior to loading into the separation column (LS column) that was attached to a magnetic stand (all from Miltenyi Biotec). After three rounds of washing, the column was removed from the magnetic stand and labeled mitochondria were eluted in storage buffer for downstream applications.

### Measurements of Complex I activity

Cell lysates were prepared using Complex I Enzyme Activity Microplate Assay Kit (Abcam), following the manufacturer’s instructions. Activity of Complex I was recorded, normalized, and quantified based on manufacturer’s instructions. Biological triplicates were performed with a minimum of three technical replicates each.

### Complex I immunoprecipitation

Complex I immunoprecipitation of MNs was prepared using Complex I Immunocapture (Abcam), following the manufacturer’s instructions. Briefly, mitochondria from MN cultures were isolated as described above and resuspended to 5.5 mg/ml in PBS with protease inhibitors. 1/10 volume of 10% of Lauryl Maltoside was added and mitochondrial membrane suspension was incubated on ice for 30 min. The suspension was centrifuged at 13,000 rpm for 10 min. The supernatant was then added to 10 μl of Complex I immunocapture beads and incubated overnight at 4 °C. Beads were washed three times with PBS/0.5% Lauryl Maltoside and eluted in SDS buffer solution. The eluted complex was analyzed by western blot analysis as described above.

### NAD^+^/NADH quantification

Purified MNs were plated onto a Matrigel pre-coated 96-well plate at 50,000 neurons per well 24 h prior to the assay. Cultures were then prepared for NAD^+^/NADH measurement using NAD^**+**^/NADH-Glo™ Assay (Promega) following manufacturer’s instructions. Levels of NAD^+^/NADH were recorded, normalized and quantified based on manufacturer’s instructions. Biological triplicates were performed with a minimum of 3 technical replicates each.

### Mitochondrial NAD^+^ quantification

Mitochondria from MN cultures (10 million cells) were isolated using the MACS technology (as described above). Mitochondrial NAD^+^ was prepared quantified using NAD^**+**^/NADH-Glo™ Assay (Promega) following manufacturer’s instructions. Briefly, isolated mitochondria were first diluted in PBS, bicarbonate base buffer, and 1% DTAB in a ratio of 2:1:1. 0.4 N of HCl was added to the mitochondrial suspension prior to heating at 60 °C for 15 min. After heating, 0.5 M of Trizma base was added to the suspension and levels of mitochondrial NAD^+^ were recorded, normalized to NAD^+^ standard curve and quantified based on manufacturer’s instructions (Promega). Biological triplicates were performed with a minimum of three technical replicates each.

### L-lactate release assay

Purified MNs were seeded at 50,000 cells per well in a 96-well plate on day 27. Culture medium was replaced with 100 μl of fresh media 2 h prior to assay. Culture medium was then collected and prepared for L-lactate measurement using L-Lactate Assay Kit (Abcam) following manufacturer’s instructions. Levels of L-lactate were recorded, normalized, and quantified based on manufacturer’s instructions. Biological triplicates were performed with a minimum of three technical replicates each.

### Lactate dehydrogenase cytotoxicity assay

Purified MNs were seeded at 50,000 cells per well in a 96-well plate on day 24. Culture medium was replaced with 200 μl of fresh media on day 25. Culture medium was then collected on days 25, 28, 31, and 35, respectively, for quantification of LDH leakage using CyQUANT™ LDH Cytotoxicity Assay (Invitrogen) following manufacturer’s instructions. Levels of leaked LDH were recorded, normalized, and quantified based on manufacturer’s instructions. Biological triplicates were performed with a minimum of three technical replicates each.

### Measurements of mitochondrial ROS levels

MN cultures at day 27 were dissociated with Accutase and seeded at 2 million cells per well in a six-well plate. At day 28, MN cultures were treated either with DMSO or 5 μM C12. After 3 days, treated MN cultures were incubated with N2B27 medium containing 5 μM MitoSOX Red (Invitrogen) for 10 min at 37 °C. The cells were then washed and analyzed by flow cytometry. Biological triplicates were performed. Flow cytometry data were analyzed using FlowJo software.

### Immunostaining of cultured cells

Cells were fixed in 4% paraformaldehyde for 15 min, permeabilized in 0.1% Triton X-100 for 15 min, and blocked in buffer containing 5% FBS and 1% BSA for an hour at room temperature. Primary antibodies (Supplementary Table S4) were diluted in blocking buffer and incubated overnight at 4 °C. Cells were washed thrice in PBS. The respective secondary antibodies were diluted 1:1500 in blocking buffer and incubated at room temperature, in the dark, for 90 min. DAPI was used at 0.1 μg/ml to visualize cellular nuclei.

### Immunohistochemistry using ALS patient tissues

Lumbar spinal cord tissue sections were cut from blocks of paraffin embedded ALS tissue (*n* = 4) and control tissue (*n* = 4), obtained from UCSD CNS biorepository. 6 μm-thick tissue sections were de-paraffinized with histology grade CitriSolv (twice for 15 min each), followed by a graded alcohol series (100, 90, 70, and 50% ethanol (vol/vol) for 3 min each), then washed in water (twice for 3 min). Endogenous peroxidase activity was then quenched in 0.6% hydrogen peroxide in methanol (vol/vol) for 15 min. After a 20 min permeabilization step in 1X PBS, 0.2% TritonX100, antigen retrieval was performed in a pressure cooker at 120 °C for 20 min in high pH solution (1% Tris based). Sections were blocked with 2% fetal bovine serum (vol/vol) and incubated with MnSOD (K68ac) antibody (1:100) overnight at 4 °C.

The following day after equilibration to room temperature, sections were washed three times in 1X PBS before 60 min room temperature incubation with 150 μl secondary antibody. Signals were detected via chromogenic reaction using NovaRed for 1–3 min per section until desired staining was achieved. Counterstaining was performed with hematoxylin for 10 s. Sections were dehydrated before adding cover slips.

### Image acquisition and image analysis

Images were acquired using the high content microscope Phenix (Perkin Elmer) using the ×20 air objective. Image analyses including cell counts and intensity measurements were performed using Columbus (Perkin Elmer).

For primary neurites analysis, neuronal projections from the soma size were determined based on SMI-32 staining. For soma size analysis, cellular nuclei were identified by DAPI, and the cytoplasmic area surrounding the nucleus was determined based on SMI-32 staining. Soma size area was measured by image analysis software (ImageJ, NIH) based on the cytoplasmic area surrounding the nucleus, excluding neuronal projections.

For patient tissues, all slides were scanned with Hamamatsu Nanozoomer 2.0HT Slide Scanner at the UCSD Microscopy Core. Using NDP.view 2 viewing software, scanned slides were evaluated at ×1 and ×20 magnifications. All neurons were evaluated in both anterior horn sections from a total of four, nonsequential tissue sections per patient, in order to ensure no overlap in neurons. K68Ac expression patterns and intensity were determined for all neurons using Fiji. Color deconvolution was performed using “H DAB” as the defined vector. Neurons were measured and quantification performed using “Colour_2” representing the VectorRed signal without the background from the counterstain (Colour_1 is hematoxylin). The region of interest was determined for each neuron and “Mean gray value” was used to quantify intensity. In order to convert intensity to optical density (OD) the formula used was: OD = log (max intensity/mean intensity) for 8-bit images. The resulting OD quantified the average darkness of the image due to DAB signal (thus representing MnSOD-K68ac stain).

### Statistical analyses

To determine the sample size for the study, a power analysis was performed based on data derived from day 35 MN survival and LDH leakage analyses. With alpha = 0.05 and power = 0.80, the projected sample size needed is a total sample size of 6 iPSC cells lines with three equal sized groups of *n* = 2 (i.e., two healthy iPSC lines, two sALS iPSC lines, and two familial ALS iPSC lines) for this between/within group comparison.

At least three biological replicates were performed for each experiment. Measurements were taken from distinct samples for analysis. Statistical analysis comparing two groups was performed by means of a two-tailed unpaired Student’s *t* test. *p* values < 0.05 were considered significant. All results are presented as mean ± standard deviation unless otherwise specified.

## Supplementary information

Supplementary Figure Legends

Supplementary Table

Supplementary Figure 1

Supplementary Figure 2

Supplementary Figure 3

Supplementary Figure 4

Supplementary Figure 5

Supplementary Figure 6

Supplementary Figure 7

## Data Availability

The authors declare that the data supporting the findings of this study are available within the paper, and its supplementary information Files. Other relevant data, if available, are available from the corresponding author(s) upon reasonable request.

## Availability of materials

Isogenic iPSCs are available from the authors, while other cell lines can be obtained from their respective sources stated in Supplementary Table S1.
