# Supplementary material for: ALS motor neurons exhibit hallmark metabolic defects that are rescued by SIRT3 activation
Source: Cell Death Differ. 2020 Nov 12;28(4):1379–97. doi: 10.1038/s41418-020-00664-0 (PMC8027637; doi:10.1038/s41418-020-00664-0)
Supplement: Supplementary file 1 — Supplementary Figure Legends [file 41418_2020_664_MOESM1_ESM.docx]

Supplemental Information

**ALS Motor Neurons Exhibit Hallmark Metabolic Defects That Are Rescued by SIRT3 Activation**

Jin-Hui Hor^1,2^, Munirah Mohamad Santosa^1,3^, Valerie Jing Wen Lim^1^, Beatrice Xuan Ho^1,2^, Amy Taylor^4^, Zi Jian Khong^1,5^, John Ravits^4^, Yong Fan^6^, Yih-Cherng Liou^2^, Boon-Seng Soh^1,2,6,#^, Shi-Yan Ng^1,3,6,7,#^

^1^ Institute of Molecular and Cell Biology, A*STAR Research Entities, Singapore 138673

^2^ Department of Biological Sciences, National University of Singapore, Singapore 117543

^3^ Yong Loo Lin School of Medicine (Physiology), National University of Singapore, Singapore 117456

^4^ Department of Neurosciences, University of California, San Diego, California, USA

^5^ School of Biological Sciences, Nanyang Technological University, Singapore 637551

^6^ The Third Affliated Hospital of Guangzhou Medical University, 510150 Guangzhou, China

^7^ National Neuroscience Institute, Singapore 308433

Correspondence to:

Shi-Yan Ng ([syng@imcb.a-star.edu.sg](mailto:syng@imcb.a-star.edu.sg))

Boon-Seng Soh ([bssoh@imcb.a-star.edu.sg](mailto:bssoh@imcb.a-star.edu.sg))

**SUPPLEMENTARY FIGURE LEGENDS**

**Supplementary Figure 1: Generation of isogenic lines using CRISPR/Cas9 technology.**

**(a)** Using established protocol, the differentiation efficiency of hiPSC towards motor neurons is around 7-10%.

Schematic summarizing the strategy for generating an isogenic knock-in for SOD1^L144F^and TDP43^G298S^ mutation and isogenic knock-out for SIRT3 into the BJ-iPS background. Guide RNA sequence is underlined with the PAM sequence highlighted in green.

**(b)** A single stranded oligonucleotide harboring the mutation serves as the repair template to facilitate the G to C transition within exon 5 that confers the leucine (L) to phenylalanine (F) mutation.

**(c)** A single stranded oligonucleotide harboring the mutation serves as the repair template to facilitate the G to A transition within exon 5 that confers the glycine (G) to serine (S) mutation.

**(d)** DNA sequencing confirms 6bp and 9bp deletion in BJ-SIRT3^+/-^ #6 and #17 exon 1 respectively.

**(e)** Both isogenic SIRT3 haploinsufficiency clones differentiates well into ISL1^+^SMI32^+^ MNs with similar efficiency as BJ-iPS. Cellular nuclei were counterstained with DAPI. Scale bars, 50 μm.

n.s. non-significant; two-tailed t test.

**Supplementary Figure 2: Metabolic flux measurements in day 10 NPCs reveal insignificant changes between ALS and healthy cells.**

**(a)** Immunostaining of unsorted BJ-iPS MN cultures at day 28 and cultures sorted with CD171 and PSA-NCAM with ISL1 and SMI32 demonstrating enrichment of ISL1^+^ MNs in the sorted cultures. Cellular nuclei were counterstained with DAPI. Scale bars, 50 μm. ***p < 0.001, n.s; two-tailed t test.

**(b)** Quantification of ISL1^+^ MN numbers in unsorted and sorted cultures at day 28 indicating more than 5-fold enrichment (from 7% to 59%).

**(c)** Quantification of total cell and ISL1^+^ MN numbers treated with 2 nM Oligomycin or 2 nM Rotenone for 3 days.

**(d)** Measurements of lactate production in culture media after 2 hours revealed increased lactate production in ALS MNs.

**(e)** OCR measurements using the MitoStress assay was performed and calculated for wild-type and ALS NPCs at day 10.

**(f)** Basal respiration, ATP production and spare respiration were calculated for NPCs from each of the cell lines reveal no significant differences in ATP production but significantly reduced spare respiration in ALS NPCs.

**(g)** ECAR measurements using the Glycolysis stress assay was performed and calculated for wild-type and ALS NPCs at day 10.

**(h)** Basal acidification, glycolysis and glycolytic capacity were calculated for NPCs from each of the cell lines reveals no significant differences in glycolysis

***p < 0.001, n.s. non-significant; One-way ANOVA, Tukey’s multiple comparisons post-hoc test.

**Supplementary Figure 3: Differentiation scheme for iPSC-derived cortical neurons and cardiomyocytes.**

**(a)** Schematic of cortical neuron differentiation protocol. Immunostaining of iPSC-derived cultures at day 28 indicating generation of BRN2^+^SATB2^+^ cortical neurons. Scale bars, 50 μm.

**(b)** Immunostaining of iPSC-derived cultures at different timepoints of cortical neuron differentiation. Cellular nuclei were counterstained with DAPI. Scale bars, 50 μm.

**(c)** Differentiation efficiency of iPSC towards cortical progenitors (FOXG1^+^) and neurons at day 14 and day 28 respectively.

**(d)** Schematic of cardiomyocyte differentiation protocol. Immunostaining of iPSC-derived cultures at day 21 indicating generation of cTnT^+^ cardiomyocytes. Cellular nuclei were counterstained with DAPI. Scale bars, 50 μm.

**Supplementary Figure 4: SIRT3 activity plays a role in ALS defective mitochondrial respiration.**

**(a)** Densitometric analyses of Western blot bands reveal no significant changes in SIRT3 protein levels in ALS versus healthy MNs. One-way ANOVA with Tukey’s multiple comparisons post-hoc test has been performed to analyze SIRT3 protein levels of wild-type and ALS MNs.

**(b)** Quantitative-PCR analysis demonstrated no significance difference in SIRT3 transcript in both WT and ALS MNs. One-way ANOVA with Tukey’s multiple comparisons post-hoc test has been performed to analyze SIRT3 transcript levels of wild-type and ALS MNs.

**(c)** Western blot analyses at day 28 confirming SIRT3 knockdown and increased MnSOD (K68ac) levels in si-SIRT3 conditions.

**(d)** Densitometric analyses of Western blot bands reveal significant increase in MnSOD (K68ac) levels in si-SIRT3 conditions.

**(e)** Measurements of basal respiration, ATP production and spare respiration of neurons in si-NT (green) and si-SIRT3 (orange) conditions.

**(f)** Western blot analyses at day 31 confirming SIRT3 overexpression in ALS MNs did not reduced MnSOD (K68ac) levels, further validating that SIRT3 activity is affected in ALS MNs.

**(g-i)** Measurements of basal respiration, ATP production and spare respiration of neurons in control (black) and SIRT3 overexpressing (grey) conditions.

*p<0.05, **p<0.01, ***p < 0.001, n.s. non-significant; two-tailed t test.

**Supplementary Figure 5: C12 activates SIRT3 and alleviates ALS diseased phenotypes**

**(a)** Western blot analyses at day 31 of BJ-SOD1^L144F^ MNs revealed a dose dependent reduction in MnSOD (K68ac) levels and no significant changes in SIRT3 protein expression as C12 concentration increased.

**(b)** Densitometric analyses of Western blot bands reveal no significant changes in SIRT3 levels and significant reduction in MnSOD (K68ac) levels in a dose dependent manner in C12 treated MNs.

**(c)** ALS MNs treated with C12 show improvement in motor neuron survival at 5µM.

**(d)** Representative images of ISL1^+^SMI32^+^ ALS MNs illustrates C12 treatment improves motor neuron survival at 5µM.

**(e-g)** Measurements of glycolysis and glycolytic capacity respectively in healthy and ALS MNs treated with DMSO as a control (light green) or 5 μM C12 (blue).

**(h)** Complex I activity in healthy and ALS MNs treated with DMSO or 5μM C12 were measured, which revealed restoration of Complex I activity in ALS MNs.

**(i)** MitoSOX analysis demonstrated reduced mitochondrial ROS in ALS MNs after C12 treatment.

*p<0.05, **p<0.01, ***p < 0.001, n.s. non-significant; two-tailed t test.

**Supplementary Figure 6: Riluzole and Edaravone do not improve neuronal morphology in ALS MNs .**

**(a-b)** Measurement of soma size and primary neurites show overall improvement in neuronal morphology in C12 treated ALS MNs.

**(c)** Representative images of ISL1^+^SMI32^+^ MNs derived from healthy and ALS iPSCs, showing cell body sizes (outlined in white dotted lines) from MNs at day 28, and at day 31 after treatment of C12. Scale bars, 50 μm.

**(d-g)** Measurement of soma size and primary neurites in Riluzole or Edaravone treated ALS MNs does not improve motor neuron morphology.

***p < 0.001, n.s. non-significant; two-tailed t test.

**Supplementary Figure 7: SIRT3 activation reversed mitochondrial respiration defects specific to ALS MNs.**

**(a)** BJ-SIRT3^+/-^ #6 and #17 were treated with either DMSO or C12 from day 28 to day 35. Number of ISL1^+^ MNs were quantified and normalized to number of ISL1^+^ MNs in respective cell lines at day 28. C12 treatment does not improve MN survival in BJ-SIRT3^+/-^.

**(b)** Measurement of soma size and primary neurites in C12 treated BJ-SIRT3^+/-^ clones does not improve motor neuron morphology. One-way ANOVA with Tukey’s multiple comparisons post-hoc test has been performed to analyze soma size and primary neurites of C12 treated WT and BJ-SIRT3^+/-^ clones MNs.

**(c)** Basal respiration, ATP production and spare respiration were calculated and revealed no significant improvement in mitochondrial bioenergetics in BJ-SIRT3^+/-^ #6 and #17 MNs.

**(d)** BJ-iPS were treated with either DMSO, FK866 or FK866 + C12 from day 28 to day 31. Number of ISL1^+^ MNs were quantified and normalized to number of ISL1^+^ MNs in respective cell lines at day 28.

**p<0.01, ***p < 0.001, n.s. non-significant; two-tailed t test.
