# Supplementary material for: ALS motor neurons exhibit hallmark metabolic defects that are rescued by SIRT3 activation
Source: Cell Death Differ. 2020 Nov 12;28(4):1379–97. doi: 10.1038/s41418-020-00664-0 (PMC8027637; doi:10.1038/s41418-020-00664-0)
Supplement: Supplementary file 2 — Supplementary Table [file 41418_2020_664_MOESM2_ESM.docx]

Supplemental Information

**ALS Motor Neurons Exhibit Hallmark Metabolic Defects That Are Rescued by SIRT3 Activation**

Jin-Hui Hor^1,2^, Munirah Mohamad Santosa^1,3^, Valerie Jing Wen Lim^1^, Beatrice Xuan Ho^1,2^, Amy Taylor^4^, Zi Jian Khong^1,5^, John Ravits^4^, Yong Fan^6^, Yih-Cherng Liou^2^, Boon-Seng Soh^1,2,6,#^, Shi-Yan Ng^1,3,6,7,#^

^1^ Institute of Molecular and Cell Biology, A*STAR Research Entities, Singapore 138673

^2^ Department of Biological Sciences, National University of Singapore, Singapore 117543

^3^ Yong Loo Lin School of Medicine (Physiology), National University of Singapore, Singapore 117456

^4^ Department of Neurosciences, University of California, San Diego, California, USA

^5^ School of Biological Sciences, Nanyang Technological University, Singapore 637551

^6^ The Third Affliated Hospital of Guangzhou Medical University, 510150 Guangzhou, China

^7^ National Neuroscience Institute, Singapore 308433

Correspondence to:

Shi-Yan Ng ([syng@imcb.a-star.edu.sg](mailto:syng@imcb.a-star.edu.sg))

Boon-Seng Soh ([bssoh@imcb.a-star.edu.sg](mailto:bssoh@imcb.a-star.edu.sg))

**SUPPLEMENTARY TABLES**

**Supplementary Table S1**: List of cell lines used

| **Cell Lines** | **Source** | **Catalog no.** |
| --- | --- | --- |
| BJ-iPS iPSC | Ng et al. (2015) | N/A |
| 18a iPSC | Eggan Lab, Boulting et al. (2011) | N/A |
| GM23720 iPSC | Coriell Institute | GM23720 |
| 29d iPSC (SOD1^L144F^) | Eggan Lab, Boulting et al. (2011) | N/A |
| 47a iPSC (TDP43^G298S^) | Eggan Lab, Rodriguez-Muela et al. (2017) | N/A |
| 19f iPSC (C9ORF72) | Eggan Lab, Kiskinis et al. (2014) | N/A |
| CS14isALS-Tn16 (sALS1) iPSC | Cedars-Sinai Medical Center’s | CS14isALS-Tn16 |
| CS51isALS-Tn3 (sALS2) iPSC | Cedars-Sinai Medical Center’s | CS51isALS-Tn3 |
| CS89isALS-Tn16 (sALS3) iPSC | Cedars-Sinai Medical Center’s | CS89isALS-Tn16 |
| BJ-SOD1^L144F^ iPSC | This paper | N/A |
| BJ-TDP43^G298S^ iPSC | This paper | N/A |
| BJ-SIRT3^+/-^ #6 iPSC | This paper | N/A |
| BJ-SIRT3^+/-^ #17 iPSC | This paper | N/A |

**Supplementary Table S2**: List of oligonucleotides used for CRISPR/Cas9 studies

| **Oligonucleotides (CRISPR)** | **Source** |
| --- | --- |
| SOD1^L144F^ sgRNA  (CACCGAGGAAACGCTGGAAGTCGTT) | Integrated DNA Technologies |
| SOD1^L144F^ ssODN  (ACATCCAAGGGAATGTTTATTGGGCGATCCCAATTACACCACAAGCGAAACGACTTCCAGCGTTTCCTGTCTTTGTACTTTCTTCATTTCCACCTTTGCC) | Integrated DNA Technologies |
| SOD1^L144F^ surveyor F:  (TAAGGGTAGCGTGTGGTGGT)  SOD1^L144F^ surveyor R:  (TGCTTAGACAAATAGGCTGTCC) | Integrated DNA Technologies |
| TDP43^G298S^ sgRNA  (CACCGTTTGGTAATAGCAGAGGGGG) | Integrated DNA Technologies |
| TDP43^G298S^ ssODN  (TTTGGGAATCAGGGTGGATTTGGTAATAGCAGAGGGGGTGGAGCTGGTTTGGGAAACAATCAAGGTAGTAATATGGGTGGTGGGATGAACT) | Integrated DNA Technologies |
| TDP43^G298S^ surveyor F  (CCACTACGCCCAGCTAATGT) | Integrated DNA Technologies |
| TDP43^G298S^ surveyor F  (TCTGGCTGGGGAATGTAGAC) | Integrated DNA Technologies |
| SIRT3^+/-^ sgRNA  (CTTCCGGCGCCGAGCGGCGCGG) | Integrated DNA Technologies |
| SIRT3^+/-^ surveyor F:  (GGCGCTCACTTCTTCGTGTA)  SIRT3^+/-^ surveyor R:  (AGACGTAGAGGCGAGTAGAGGA) | Integrated DNA Technologies |

**Supplementary Table S3**: List of human primers used in qPCR studies

| **Oligonucleotides (qPCR)** | **Source** |
| --- | --- |
| CHOP qPCR F:  (AAGGCACTGAGCGTATCATGT)  CHOP qPCR R:  (TGAAGATACACTTCCTTCTTGAACA) | Ng et al. (2015) |
| sXBP1 qPCR F:  (TGCTGAGTCCGCAGCAGGTG)  sXBP1 qPCR R:  (GCTGGCAGGCTCTGGGGAAG) | Ng et al. (2015) |
| SIRT3 qPCR F:  (CCCTGGAAACTACAAGCCCAAC)  SIRT3 qPCR R:  (GCAGAGGCAAAGGTTCCATGAG) | This paper |
| ACTB qPCR F:  (CCAACCGCGAGAAGATGA)  ACTB qPCR R:  (CCAGAGGCGTACAGGGATAG) | Ng et al. (2015) |
| HPRT qPCR F:  (TATGGCGACCCGCAGCCCT)  HPRT qPCR R:  (CATCTCGAGCAAGACGTTCAG) | This paper |

**Supplementary Table S4**: List of antibodies used in western blot and immunostaining studies

| **Antibodies** | **Source** | **Catalog** |
| --- | --- | --- |
| Rabbit anti-SirT3 (D22A3) | Cell Signaling | 5490 |
| Mouse anti-alpha tubulin (B-7) | Santa Cruz | sc-5286 |
| Mouse anti-TOMM20 | Abcam | ab56783 |
| Rabbit anti-Islet 1 [EP4182] | Abcam | ab109517 |
| Mouse anti-SMI-32 | BioLegend | 801701 |
| Rabbit anti-Acetylated-Lysine | Cell Signaling | 9814 |
| Rabbit anti-Acetylated-Lysine | Abcam | ab21623 |
| Mouse anti-NDUFA9 antibody [20C11B11B11] | Abcam | ab14713 |
| Mouse anti-UQCRC2 antibody [13G12AF12BB11] | Abcam | ab14745 |
| Rabbit anti- SDHA (D6J9M) | Cell Signaling | 11998 |
| Rabbit anti-COXIV (3E11) | Cell Signaling | 4850 |
| Rabbit anti-ATPB | Abcam | ab14730 |
| Rabbit anti-SOD2/MnSOD | Abcam | ab13533 |
| Rabbit anti- SOD2/MnSOD (acetyl K68) | Abcam | ab137037 |
| Rabbit anti-SATB2 [EPNCIR130A] | Abcam | ab92446 |
| Goat anti-BRN2 (C-20) | Santa Cruz | sc-6029 |
| Rabbit anti-FOXG1 | Abcam | ab18259 |
| Rabbit anti-SOX1 | Abcam | ab87775 |
| Mouse anti-NESTIN [10C2] | Abcam | ab22035 |
| Rabbit anti-Doublecortin | Abcam | ab18723 |
| Mouse anti-TUJ1 | BioLegend | 801201 |
| Mouse anti-Troponin T | Thermo Fisher Scientific | MS-295-P |
| CD171 (L1CAM)-APC | Miltenyi Biotec | 130-100-684 |
| Anti-PSA-NCAM-APC | Miltenyi Biotec | 130-120-437 |
| AlexaFluor Donkey anti-Mouse 488 | Thermo Fisher Scientific | A21202 |
| AlexaFluor Donkey anti-Rabbit 647 | Thermo Fisher Scientific | A31573 |
| AlexaFluor Donkey anti-Rabbit 488 | Thermo Fisher Scientific | A21206 |
| AlexaFluor Donkey anti-Mouse 568 | Thermo Fisher Scientific | A10037 |
| Goat anti-rabbit IgG, HRP conjugated | Thermo Fisher Scientific | 31466 |
| Goat anti-mouse IgG, HRP conjugated | Thermo Fisher Scientific | 31431 |
