# Supplementary figures and images for: ALS motor neurons exhibit hallmark metabolic defects that are rescued by SIRT3 activation
Source: Cell Death Differ. 2020 Nov 12;28(4):1379–97. doi: 10.1038/s41418-020-00664-0 (PMC8027637; doi:10.1038/s41418-020-00664-0)

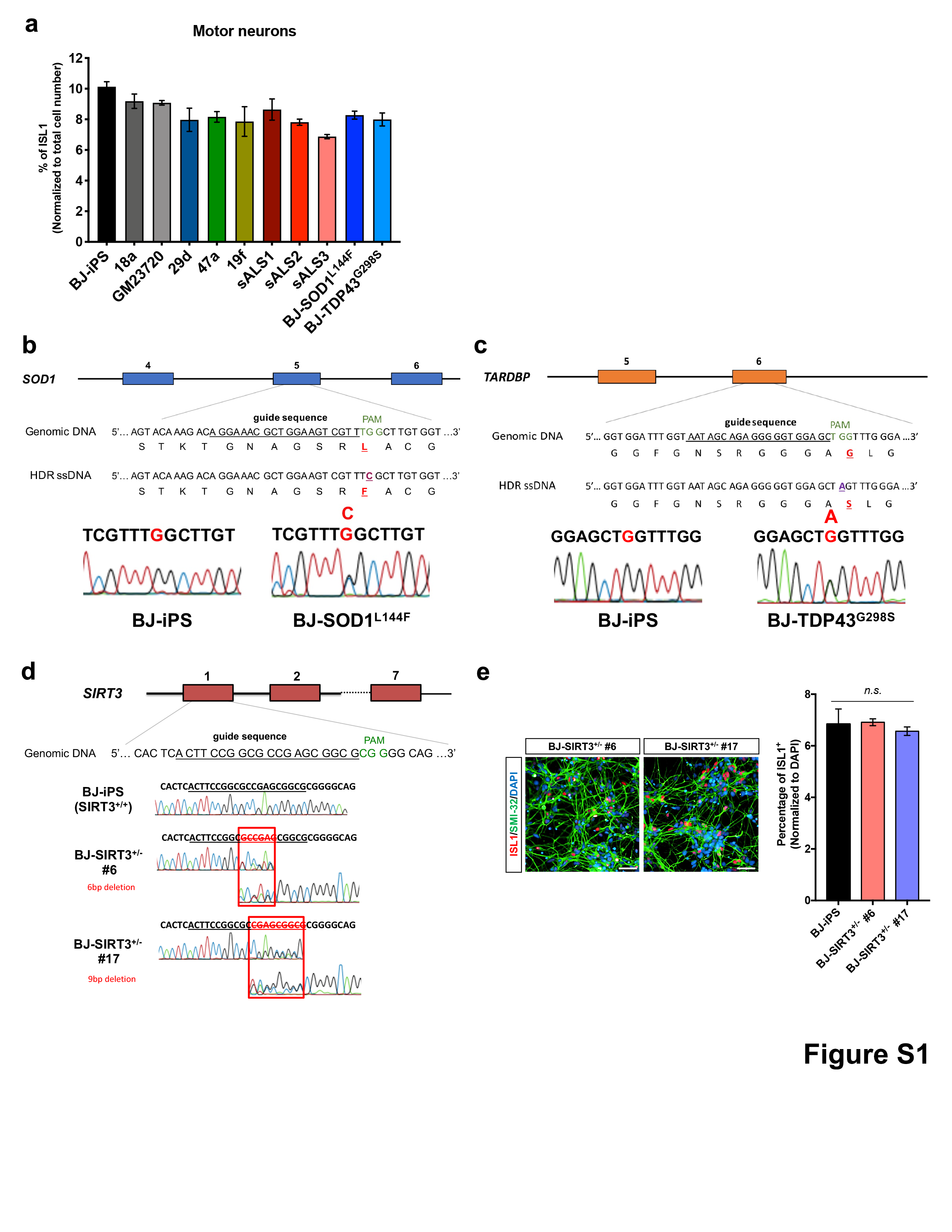

Supplement: Supplementary file 3 — Supplementary Figure 1 [file 41418_2020_664_MOESM3_ESM.png]

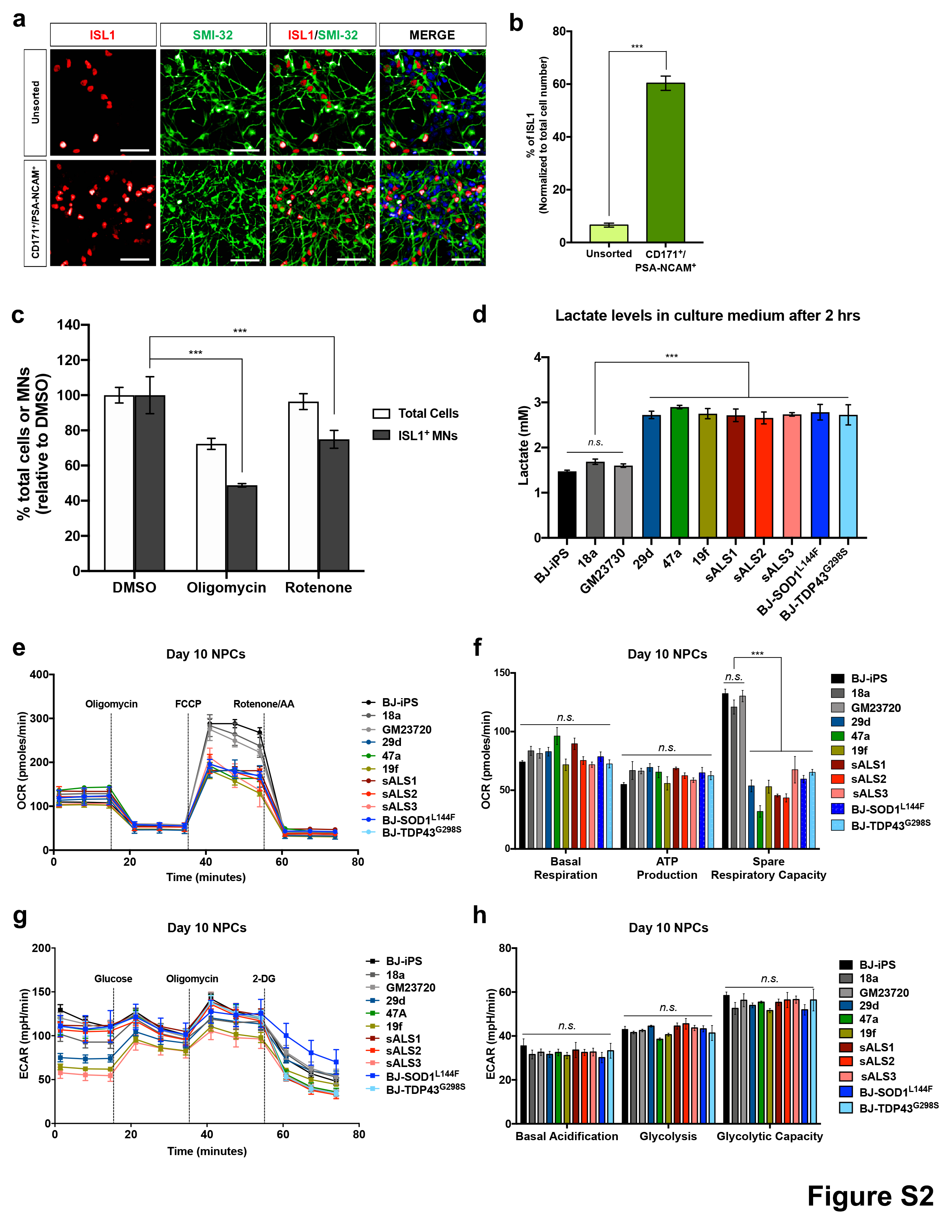

Supplement: Supplementary file 4 — Supplementary Figure 2 [file 41418_2020_664_MOESM4_ESM.png]

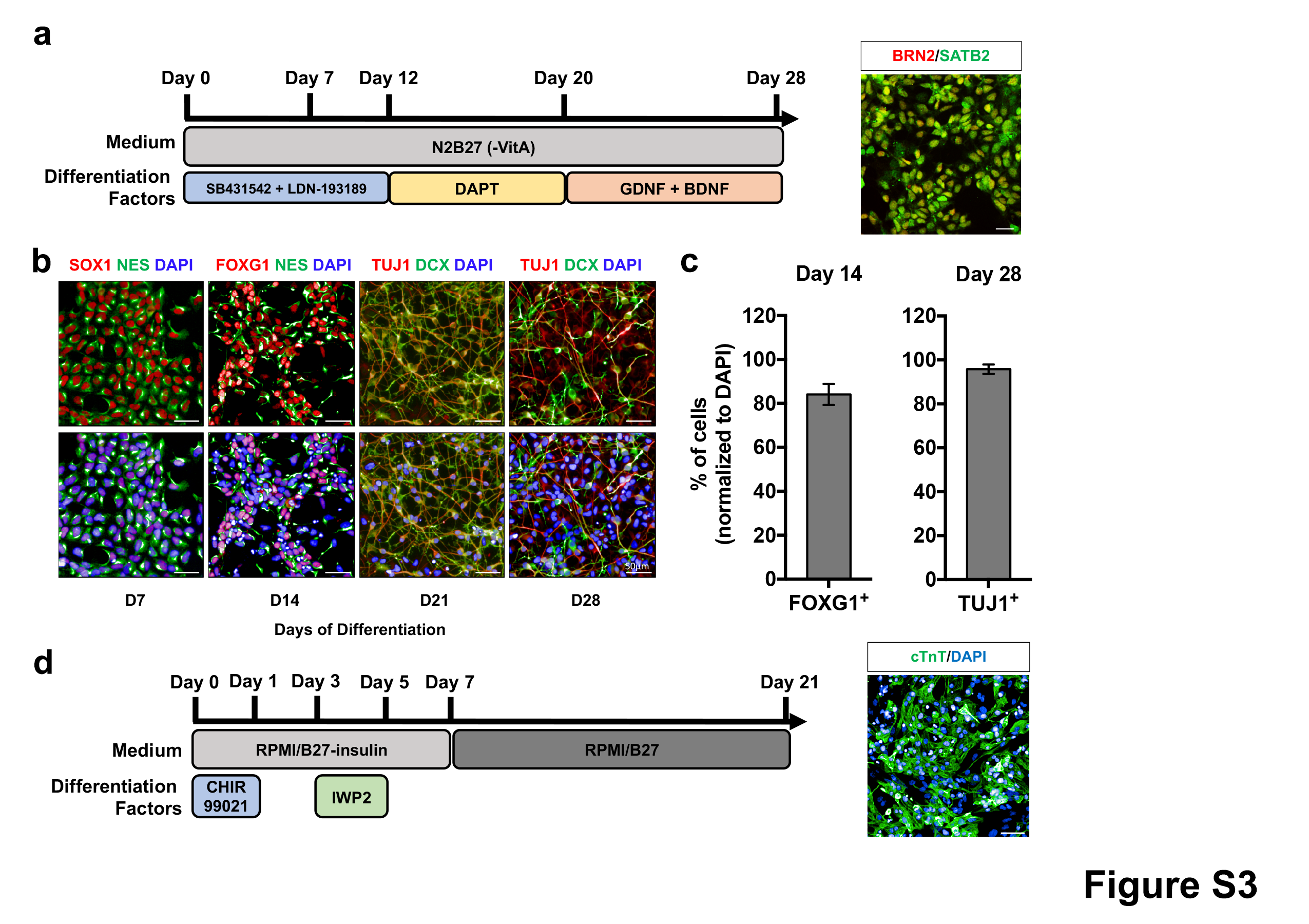

Supplement: Supplementary file 5 — Supplementary Figure 3 [file 41418_2020_664_MOESM5_ESM.png]

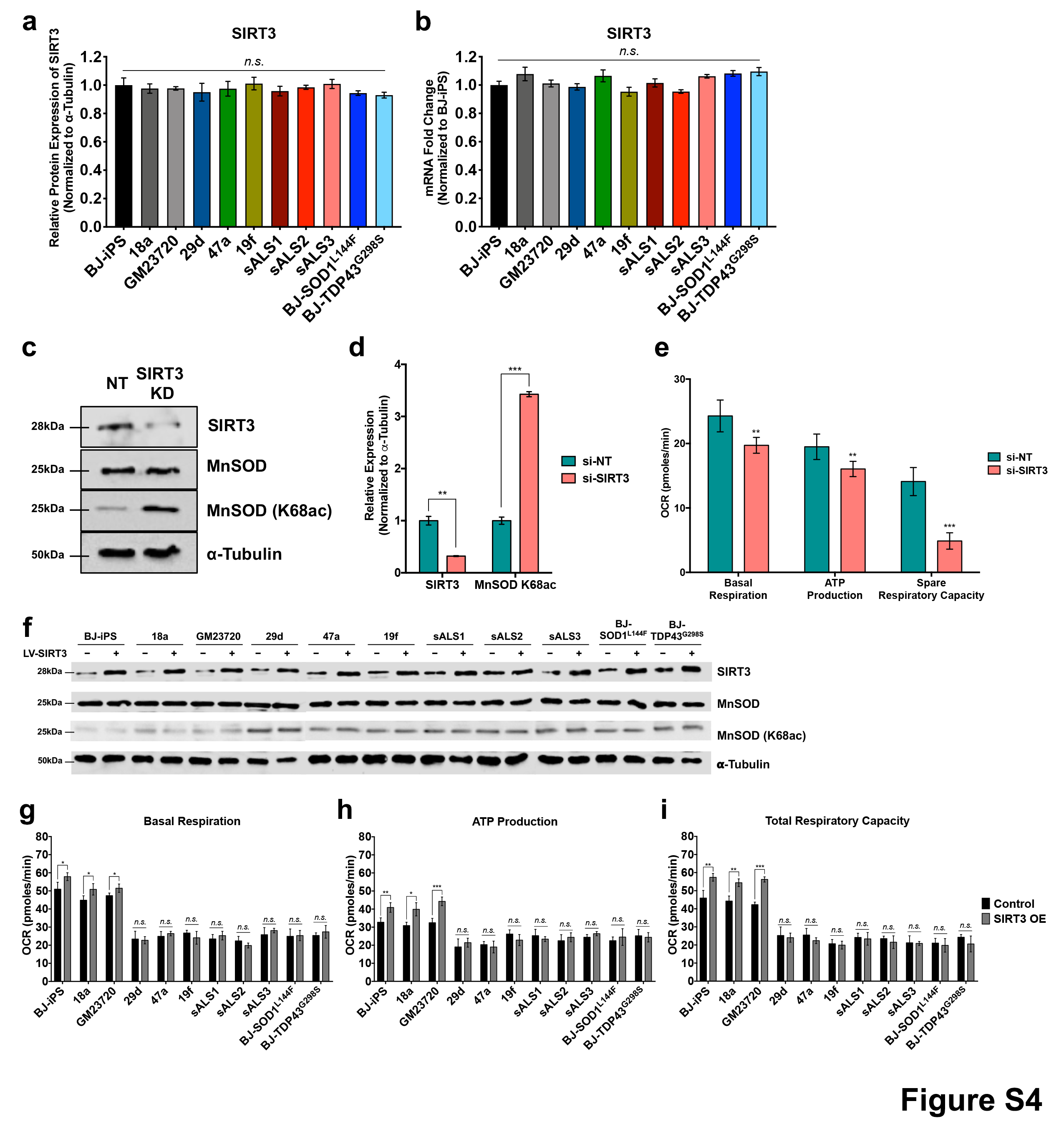

Supplement: Supplementary file 6 — Supplementary Figure 4 [file 41418_2020_664_MOESM6_ESM.png]

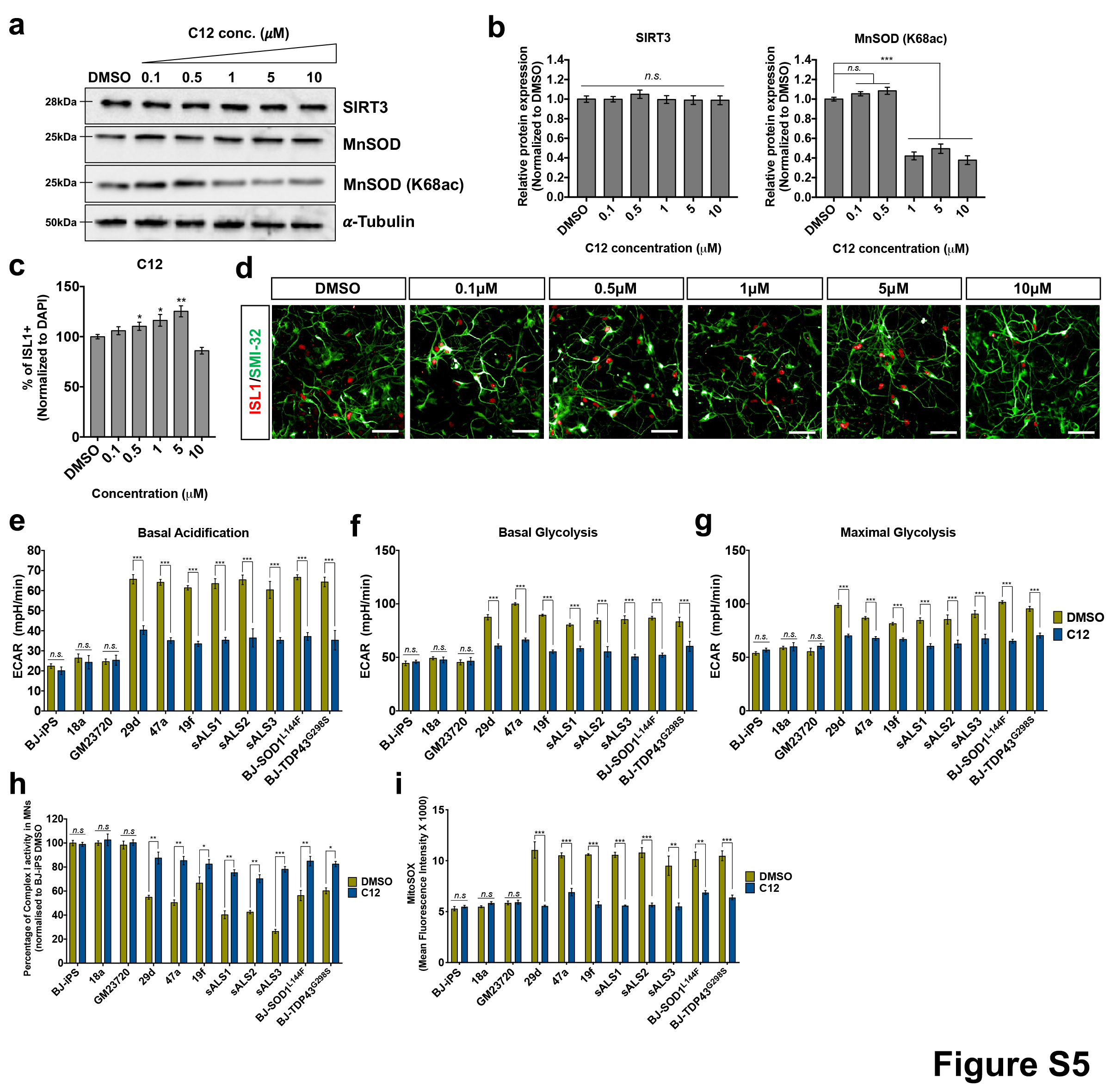

Supplement: Supplementary file 7 — Supplementary Figure 5 [file 41418_2020_664_MOESM7_ESM.png]

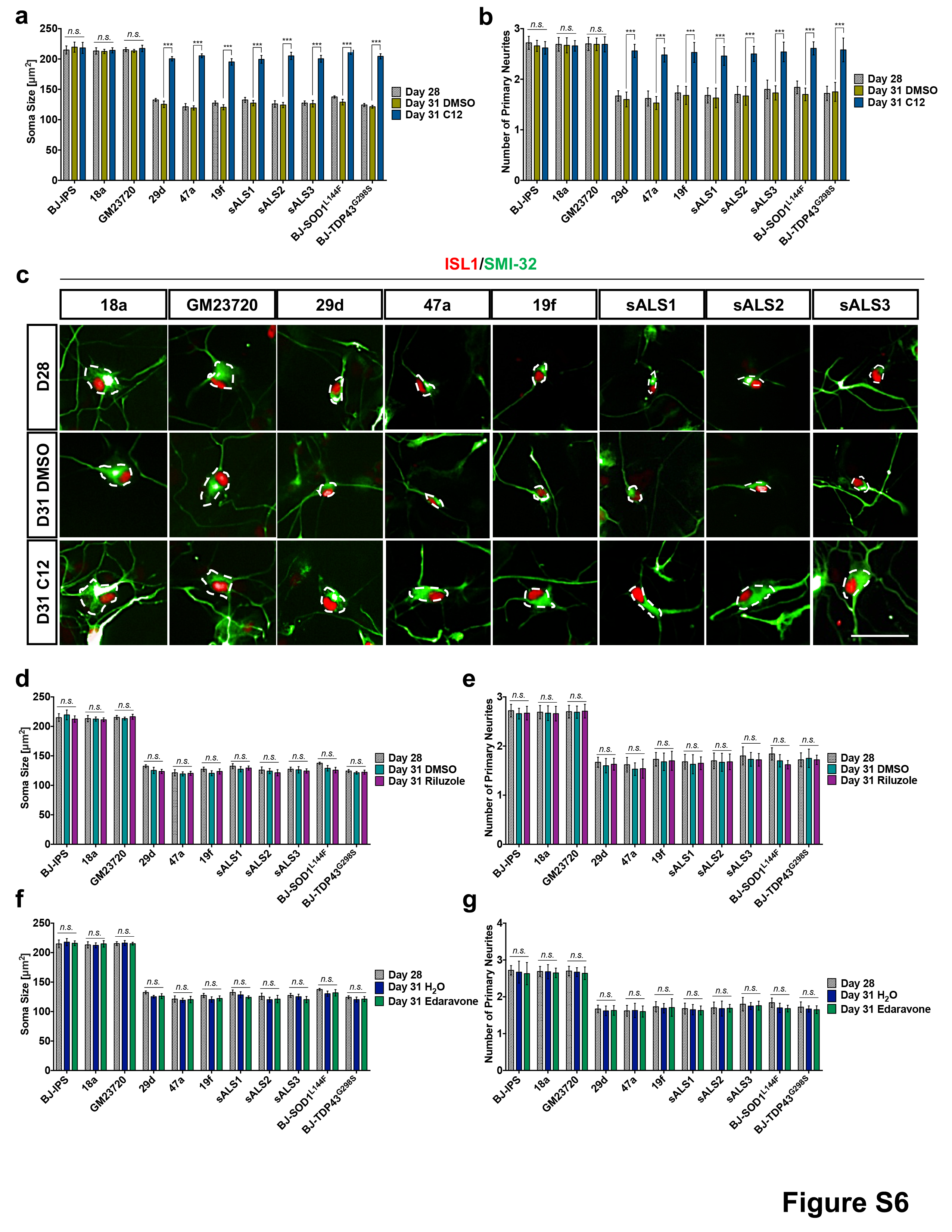

Supplement: Supplementary file 8 — Supplementary Figure 6 [file 41418_2020_664_MOESM8_ESM.png]

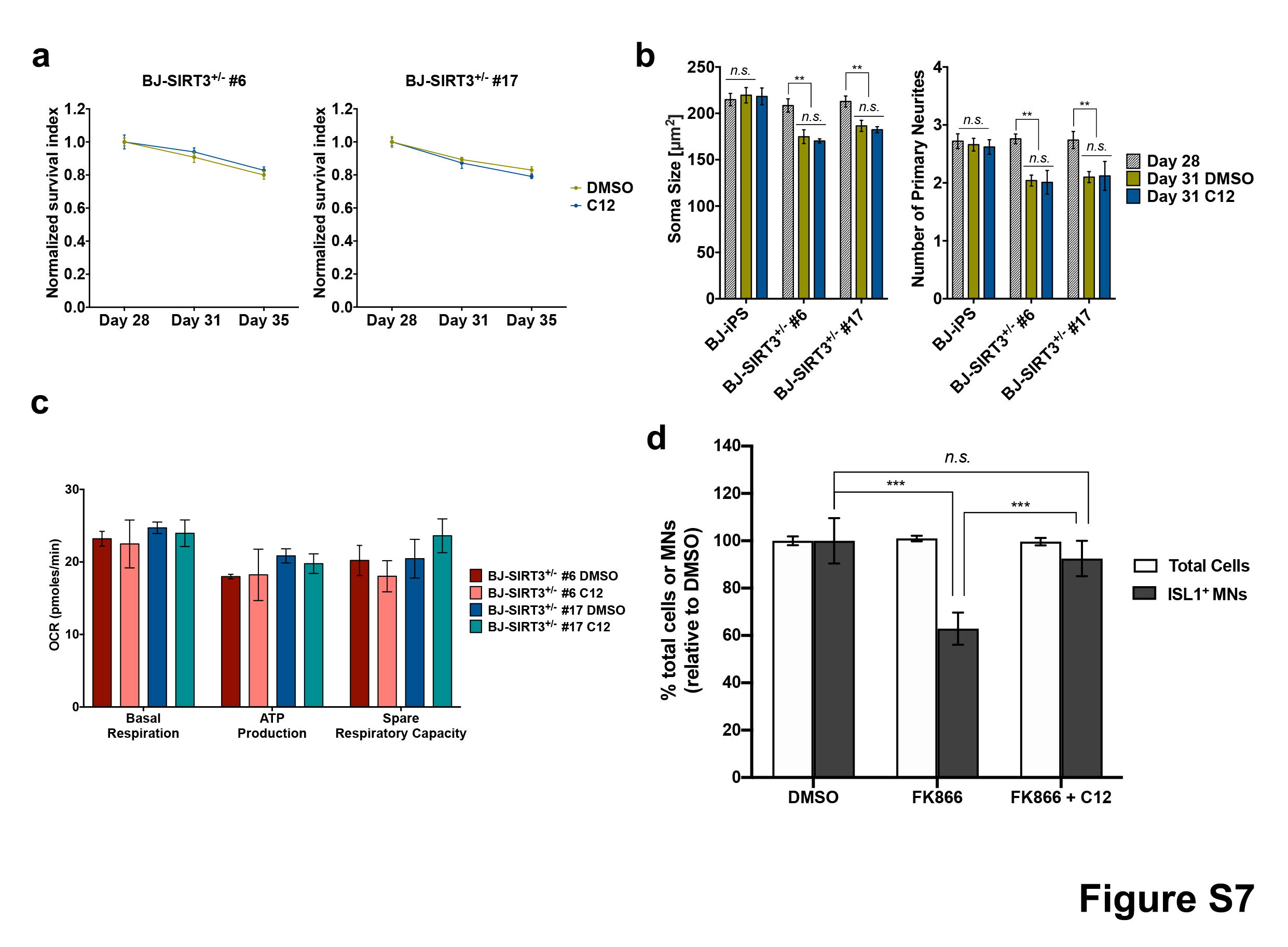

Supplement: Supplementary file 9 — Supplementary Figure 7 [file 41418_2020_664_MOESM9_ESM.png]
